# Emerging Technologies in Cardiovascular Disease Modeling: Bridging Basic Science and Clinical Translation

**DOI:** 10.3390/cells15191754

**Published:** 2026-09-26

**Authors:** Ramcharan Singh Angom, Sophie K. Ashbrook, Carter Magnano, Riya Kar, Anya Dorairaj, Hari Krishnareddy Rachamala, Debabrata Mukhopadhyay

**Affiliations:** 1 Department of Biochemistry & Molecular Biology, Mayo Clinic College of Medicine and Sciences, Jacksonville, FL 32224, USA; 2 Department of Biological Engineering, Massachusetts Institute of Technology, Cambridge, MA 02139, USA

**Keywords:** cardiovascular disease modeling, human induced pluripotent stem cells, cardiac organoids, tissue engineering, organ-on-chip, CRISPR genome editing, multi-omics, artificial intelligence, precision medicine

## Abstract

Cardiovascular diseases (CVDs) remain the leading cause of death worldwide, yet therapeutic development is limited by experimental models that incompletely recapitulate human physiology, disease heterogeneity, and treatment responses. Emerging human-relevant models, including patient-derived induced pluripotent stem cells, cardiac organoids, engineered tissues, organ-on-chip systems, genome editing, multi-omics, and computational approaches, are improving the ability to investigate cardiovascular disease mechanisms and therapeutic responses. In this review, we critically compare these technologies with respect to physiological relevance, reproducibility, scalability, throughput, and translational potential. We highlight their applications in disease modeling, drug discovery, toxicity assessment, and precision medicine while examining persistent limitations, including cellular immaturity, incomplete vascularization and systemic integration, model variability, and limited clinical validation. We further discuss how integration of bioengineering, genome editing, multi-omics, and computational methods may improve predictive performance. Standardization, interlaboratory validation, regulatory qualification, and prospective benchmarking against human clinical outcomes will be essential for translating these platforms into reliable tools for cardiovascular research and therapeutic development. Collectively, the convergence of stem cell biology, bioengineering, genome editing, multi-omics, and computational approaches is establishing a new generation of human-centered cardiovascular models with the potential to strengthen mechanistic discovery and accelerate the development of more predictive and personalized therapies.

## 1. Introduction

### 1.1. Global Burden of Cardiovascular Diseases

Cardiovascular diseases (CVDs) remain the leading cause of mortality and a major contributor to disability and healthcare expenditure worldwide [1]. Recent estimates from the Global Burden of Disease study indicate that approximately 19.2 million deaths were attributable to CVDs in 2023, while the number of prevalent cases exceeded 626 million globally [2]. Despite substantial advances in prevention, diagnosis, pharmacological management, and interventional cardiology [3], the absolute burden of CVD continues to increase, driven in part by population growth and population aging [4], and persistent exposure to metabolic, behavioral, and environmental risk factors [5,6]. Importantly, cardiovascular disease burden is not distributed equally across populations. Low- and middle-income countries account for a disproportionate share of cardiovascular deaths, while substantial differences in disease incidence, treatment access, and clinical outcomes are also observed according to sex, race and ethnicity, socioeconomic status, and geographic location [7,8]. These disparities emphasize the need for experimental systems capable of representing not only general mechanisms of cardiovascular pathology but also interindividual and population-level heterogeneity (Figure 1).

Experimental models that reproduce only isolated components of cardiovascular physiology may not capture clinically important mechanisms of disease progression or therapeutic response [9,10]. Developing human-relevant experimental platforms that reproduce this cellular, structural, mechanical, and genetic complexity has therefore become a major priority in cardiovascular research.

### 1.2. Limitations of Traditional Cardiovascular Models

Conventional two-dimensional cultures provide experimentally accessible and scalable systems but lack the multicellular architecture, extracellular matrix interactions, mechanical forces, and physiological gradients of native cardiovascular tissues. Animal models provide systemic complexity but are limited by interspecies differences in electrophysiology, metabolism, immune responses, vascular biology, and disease progression. These limitations contribute to persistent gaps between preclinical findings and clinical translation [10,11,12]. Species ranging from zebrafish and rodents to pigs, sheep, and non-human primates have been used to model cardiovascular development and disease [13]. Nevertheless, important interspecies differences exist in cardiac anatomy, heart rate, electrophysiology, metabolism, immune responses, vascular biology, regenerative capacity, and gene regulation [14,15]. These limitations become particularly important when experimental findings are translated into therapeutic development. Compounds demonstrating efficacy or acceptable toxicity in conventional preclinical models do not necessarily produce equivalent responses in humans. Moreover, genetically homogeneous cell lines and laboratory animals inadequately represent substantial genetic and phenotypic heterogeneity present among patients [9,16]. Traditional models, therefore, remain essential but are increasingly complemented by experimental systems designed to incorporate human genetic backgrounds, multicellular organization, tissue-specific microenvironments, and physiologically relevant mechanical and biochemical cues.

### 1.3. Emergence of Next-Generation Cardiovascular Disease Models

Rapid advances in stem-cell biology, tissue engineering, genome editing, microfabrication, omics technologies, and computational science have therefore enabled the development of a new generation of cardiovascular disease models (Figure 2). Rather than relying exclusively on simplified cell monolayers or cross-species extrapolation, these platforms aim to reproduce specific components of human cardiovascular physiology with greater biological and clinical relevance [17].

These include patient-derived hiPSC models, cardiac organoids and spheroids, engineered heart tissues, biomaterial and bioprinting platforms, organ-on-chip systems, CRISPR-based genome engineering, and computational approaches. Rather than replacing conventional models, these technologies provide complementary levels of biological complexity and experimental control. This review critically evaluates their strengths, limitations, translational applications, and suitability for different cardiovascular research questions [18,19].

Organ-on-chip and microphysiological systems provide another level of experimental control by combining living human cells with microfluidic engineering [20,21]. Integration of cardiac tissues with vascular, hepatic, renal, immune, or other organ-specific compartments may further enable investigation of systemic drug responses and multiorgan pathophysiology [21,22,23]. Concurrently, advances in biomaterials and three-dimensional bioprinting have improved the ability to reproduce extracellular matrix properties and spatially organize multiple cardiovascular cell populations [24]. These experimental advances are increasingly being integrated with single-cell and spatial transcriptomics, proteomics, metabolomics, high-content imaging, and artificial intelligence (AI). Multimodal datasets can characterize cardiovascular disease at unprecedented cellular and molecular resolution, while computational approaches can identify disease-associated signatures, classify phenotypes, predict drug responses, and assist in interpreting increasingly complex experimental systems [25,26]. In this context, next-generation cardiovascular models should not necessarily be viewed as replacements for all conventional experimental systems. Rather, they represent complementary platforms that can bridge mechanistic studies and clinical translation by providing increasingly human-relevant experimental environments.

### 1.4. Scope and Objectives of This Review

The rapid expansion of human-based cardiovascular modeling technologies has created a diverse experimental landscape ranging from patient-derived cellular models to multicellular tissues, microphysiological systems, genome-edited platforms, and computational approaches [27,28,29]. Each model captures distinct aspects of cardiovascular physiology and disease, and no single system currently reproduces the full structural, cellular, mechanical, electrophysiological, vascular, immune, and systemic complexity of the human cardiovascular system [29,30]. The central challenge is therefore not simply to find the most sophisticated model, but to determine which platform-or combination of platforms-is most appropriate for a specific biological, pharmacological, or translational question.

This review examines emerging technologies that are reshaping cardiovascular disease modeling and therapeutic development. We first discuss hiPSC-based cardiovascular models, including patient-specific cardiomyocytes and vascular cell populations, and their applications in genetic disease modeling, mechanistic investigation, drug screening, and precision medicine [31]. We then examine three-dimensional cardiac spheroids and organoids, with emphasis on their cellular composition, self-organization, disease-modeling applications, and current limitations [32,33]. Cardiac tissue-engineering strategies are then considered, including natural and synthetic biomaterials, decellularized extracellular matrices, engineered heart tissues, and three-dimensional bioprinting approaches to recreate the structural and biomechanical properties of native myocardium.

The review further evaluates organ-on-chip and microphysiological platforms capable of incorporating perfusion, shear stress, mechanical stimulation, and inter-organ communication, as well as genome-editing approaches that permit causal interrogation of cardiovascular disease-associated genes and variants [34]. Finally, we consider how single-cell and spatial omics, high-content phenotyping, computational modeling, and AI can be integrated with experimental cardiovascular platforms to improve disease classification, therapeutic screening, toxicity prediction, and patient-specific treatment selection [33,35]. Emphasis is placed on the relative strengths and limitations of each platform, including physiological relevance, cellular maturity, reproducibility, scalability, throughput, cost, and compatibility with precision-medicine applications [30,36]. We also discuss persistent barriers to clinical translation, including model standardization, validation against human tissues and clinical outcomes, incorporation of vascular, immune, neural, and systemic components, and establishment of regulatory frameworks for next-generation preclinical models [20,36]. Collectively, these emerging technologies represent a transition from reductionist cardiovascular models toward integrated, human-specific experimental systems that may improve mechanistic understanding, enhance prediction of therapeutic efficacy and toxicity, and ultimately facilitate the development of more personalized cardiovascular therapies.

## 2. Human iPSC Models

### 2.1. Generation and Differentiation of iPSCs

Human induced pluripotent stem cells (hiPSCs) can be generated from somatic cells and differentiated into cardiovascular cell types, including cardiomyocytes, endothelial cells, vascular smooth muscle cells, pericytes, and fibroblasts [37,38]. A major advantage of iPSC technology is the ability to generate renewable, patient-specific pluripotent cell populations that retain the donor’s genetic background and can subsequently be differentiated into a wide range of specialized cell types [39]. Human iPSCs were first generated by Takahashi and colleagues in 2007 through ectopic expressions of OCT4, SOX2, KLF4, and c-MYC, collectively known as the Yamanaka factors [38]. This technology addressed important limitations associated with ESCs, particularly ethical concerns surrounding embryo-derived cells, while enabling the generation of patient-specific pluripotent cell lines [40]. Human dermal fibroblasts are among the most commonly used somatic cell sources for iPSC generation and can be obtained through skin punch biopsy [41,42]. During reprogramming, cells progressively lose somatic-cell transcriptional programs while sequentially activating early and late pluripotency-associated gene networks [43]. Their principal advantage for disease modeling is the ability to retain the donor’s genetic background while providing renewable human cell populations. Non-integrating reprogramming approaches, including Sendai virus, episomal vectors, and modified mRNA, are now commonly used to generate hiPSC lines. Cardiovascular differentiation is achieved through controlled modulation of developmental signaling pathways, particularly Wnt/β-catenin signaling for cardiomyocyte generation [39,44,45,46]. Following successful reprogramming, iPSCs can be differentiated toward specific cell lineages by controlling developmental signaling pathways. Cells are first directed toward ectodermal, mesodermal, or endodermal progenitor states and subsequently exposed to lineage-specific signals that promote differentiation toward the desired mature cell population [47]. Patient-derived hiPSC cardiovascular cells have become important tools for investigating genotype–phenotype relationships, disease mechanisms, drug toxicity, and interindividual therapeutic responses. Their major limitations include cellular immaturity, donor- and line-dependent variability, and incomplete reproduction of multicellular and systemic physiology.

### 2.2. iPSC-Derived Cardiomyocytes

Cardiomyocytes are the electrically excitable, contractile cells responsible for generating the force required for cardiac contraction. One of the most widely used approaches for differentiating hiPSCs into cardiomyocytes involves biphasic modulation of canonical Wnt/β-catenin signaling. During early differentiation, Wnt signaling is activated, commonly using a glycogen synthase kinase-3 (GSK3) inhibitor such as CHIR99021, to promote mesoderm formation. Subsequent inhibition of Wnt signaling promotes cardiac lineage specification [45]. Patient-specific iPSC-derived cardiomyocytes (iPSC-CMs) provide an opportunity to investigate cardiovascular disease mechanisms and pharmacological responses within the context of an individual’s genetic background. These cells express major cardiac structural proteins and ion channels and reproduce numerous disease-associated electrophysiological, contractile, and calcium-handling phenotypes. Consequently, patient-derived iPSC-CMs have become valuable platforms for studying genotype–phenotype relationships, drug toxicity, and interindividual variation in therapeutic response [19] (Table 1).

#### 2.2.1. Cardiomyopathy

Cardiomyopathy is a diverse categorization of heart disease that involves structural dysfunction. The most common form of cardiomyopathy is dilated cardiomyopathy (DCM), which is a condition where one or both of the ventricles have become less contractile with enlargement of the ventricular chamber [48]. Hearts affected by DCM frequently experience systolic congestive heart failure, marked by ventricular dilatation and reduced inotropy, often producing systemic hypotension. Moreover, patients with DCM often experience potentially life-threatening ventricular arrhythmias due to electrical dysfunction [49]. This electrical dysfunction may be caused directly by ion channel abnormalities, but also through interstitial fibrosis, disarray in the cellular arrangement of cardiomyocytes, and abnormal connections between cardiomyocytes [50]. One common genetic cause of DCM is a truncating mutation in the Titin (TTN), accounting for roughly 30% of DCM patients [51]. Gramlich et al. generated iPSC-CMs with truncating TTN mutations and applied antisense oligonucleotides (AONs) to skip the TTN truncation in Exon 326, which led to improved myofibril assembly and stability of the sarcomere. This phenotypic rescue was replicated in animal models [51]. Hypertrophic cardiomyopathy (HCM) is a cardiac disorder characterized by left ventricular hypertrophy, causing the left ventricle to become too thick and stiff, impairing its ability to pump blood effectively. This disease is a common cause of sudden cardiac arrest in young athletes [52]. HCM commonly results from mutations occurring in genes encoding sarcomeric proteins, such as MYH7 and MYBPC3, which account for roughly half of inherited HCM cases [52]. HCM was simulated in vitro by Cohn et al., who were able to induce mutations in the MYH7 and MYBPC3 genes via CRISPR, which generated iPSC-CMs with hypercontractibility, increased tension, and delayed reaction kinetics, exactly what would be expected in an in vivo model [53].

#### 2.2.2. Arrhythmias

Arrhythmias are disorders of the electrical impulse formation and/or conduction in cardiomyocytes. Long QT Syndrome (LQTS) arises when there is delayed repolarization of the cardiomyocyte action potential, leading to prolongation of the QT interval on the electrocardiogram [46]. Itzhaki et al. found that iPSC-CMs generated from a patient with Type II LQTS, which is characterized by a decrease in the outward potassium ion current, displayed a 60–72% reduction in Ikr in vitro, compared to a negative control. This reduction in the repolarization current is comparable to that seen in human LQTS II phenotypes [54]. Catecholaminergic polymorphic ventricular tachycardia (CPVT) is an inherited arrhythmia syndrome characterized by adrenergic-triggered ventricular arrhythmias in the absence of major structural heart disease. Many cases are associated with pathogenic variants in RYR2, which can disrupt sarcoplasmic-reticulum calcium handling. Patient-derived iPSC-CMs carrying CPVT-associated variants recapitulate abnormal calcium release and trigger electrical activity in response to adrenergic stimulation, whereas control cells do not show the same phenotype [55]. These findings show the utility of iPSC-CMs for reproducing patient-specific abnormalities in excitation-contraction coupling.

#### 2.2.3. Ischemic Heart Disease

Ischemic heart disease (IHD) is the most common form of heart disease worldwide and a significant cause of heart failure. IHD is caused by insufficient perfusion of oxygen-rich blood to the myocardium to meet metabolic demands, causing cell damage and death. Upon reperfusion, which is necessary for oxygen restoration, damage is compounded through a distinct secondary injury cascade [56]. iPSC-CMs have been developed to model IHD. IPSC-CMs are subjected to hypoxia via hypoxia chambers, which damage sarcomeres and induce cell death, thereby mimicking typical cardiac responses. Following hypoxia, iPSC-CMs have displayed multiple electrophysiological abnormalities, including reduced automaticity, prolonged depolarization time, shortened field potential duration and slowed conduction velocity, all of which were reversible on reoxygenation [56]. Reperfusion-specific damage was notable upon reoxygenation, as iPSC-CMs experienced reductions in both sarcomeric protein expression (MyBPC3, Troponin T) and the calcium-handling gene SLC8A1, to a greater extent than with hypoxia alone. A broader panel of metabolic, sarcomeric, and calcium-handling genes (ACADM, PFKM, TNNT2, MYBPC3, MAP4K4, RYR2, ATP2A2) also showed significant decreases following the 24h hypoxia-reoxygenation, not seen with hypoxia alone [56].

#### 2.2.4. Heart Failure

Heart failure (HF) is the inability of the heart to circulate sufficient blood throughout the body. Heart failure can be characterized by congestive symptoms of fluid backup, hypoperfusion of systemic organs due to impaired pumping function, or both [57]. To mimic HF, Bissoli et al. administered endothelin-1 (ET-1) to iPSC-CMs, which induced features common in HF, including increased stress markers (ANP/NPPA, BNP/NPPB, ACTA1), decreased contractile force, increased expression of fibrotic remodeling factors, and protein aggregation, indicating impaired proteostasis. In this study, 3D organoids exhibited a stronger induction of these markers relative to the monolayer iPSC-CMs, suggesting that multi-cell-type 3D architecture may better recapitulate the physiological complexity of heart failure [58]. A considerable limitation of iPSC-CMs is that their phenotypes resemble those of embryos rather than mature cells, leading to disorganized morphology, reduced contractile capacity, altered glycolytic metabolism, and abnormal electrophysiological properties [52]. Immature CMs have a metabolism reliant on glycolysis, being more sensitive to reduced glucose than oxygen, making immature CMs artificially resistant to ischemic reperfusion damage [56,59]. Hidalgo et al. were able to mature hPSC-CMs by culturing them for 8 days in glucose-free, galactose and fatty acid-supplemented medium to force a metabolic shift from glycolysis to mitochondrial fatty-acid oxidation [59]. Although iPSC-derived CMs have transformed the study of cardiac disease mechanisms and drug responses, many cardiovascular disorders also involve the vasculature, driving the development of iPSC-derived vascular cell models.

### 2.3. iPSC-Derived Vascular Cells

#### 2.3.1. Endothelial Cells

Endothelial cells form the innermost lining of lymphatic and vascular tissues, serving as a selective barrier between the bloodstream and surrounding tissues. Endothelial cells are critical for transporting molecules across vessel walls and regulating blood flow in response to signaling molecules such as nitric oxide, adrenaline, and angiotensin II [60]. HUVECs, derived from fetal umbilical cords and differing in gene expression from microvascular endothelial cells, are currently used as endothelial cell lines in 2D culture systems [61]. Because of this, the generation of vascular endothelial cells is crucial for research efforts. Generating iPSC-ECs consists of first deriving the pluripotent cells into mesoderm cells by inhibiting GS3K and adding BMP4, then adding VEGF-A to direct cells to endothelial cells and selecting for endothelial cells via surface marker sorting [62]. To direct endothelial cells toward arterial or venous phenotypes, defined patterning cues must be applied at the endothelial precursor stage rather than left to default differentiation. Graded VEGF signaling, acting upstream of Notch, imposes arterial identity, while lower VEGF permits NR2F2 (COUP-TFII)-driven venous identity [63]. In the basal state, endothelial cells exhibit low ICAM-1 expression, a protein that binds leukocytes to endothelial cells. Endothelial cells produce Nitric Oxide (NO), which relaxes vascular smooth muscle cells, thereby dilating blood vessels and maintaining low vascular permeability. Under stressed conditions, ICAM-1 expression is increased, NO production decreases, and endothelial cell permeability is increased, a condition commonly referred to as endothelial dysfunction [64,65,66].

Familial pulmonary arterial hypertension (FPAH) is a progressive vascular disease frequently associated with mutations in the BMPR2 gene. When modeled in vitro, patient-derived iPSC-ECs successfully recapitulate the cellular hallmarks of the FPAH-specific endothelial dysfunction. Compared to healthy control ECs, FPAH iPSC-ECs display compromised cell adhesion to extracellular matrices, reduced survival, impaired cell migration, and disordered angiogenesis. The current hurdle in understanding FPAH is its partial penetrance, as only about 20% of individuals carrying the BMPR2 mutation develop the disease [67]. Familial hypercholesterolemia (FH) is caused by mutations in the LDLR gene, a condition in which high cholesterol is known to damage ECs through inflammation and oxidative stress. Zakharova et al. investigated whether FH patients’ ECs also carried an inherent LDLR defect, generating iPSC-ECs from FH patients and finding reduced LDL uptake and lower levels of mature LDLR compared to control iPSC-ECs, prior to any cholesterol exposure. This separates two previously conflated sources of endothelial damage: cholesterol-driven injury to an otherwise normal endothelium versus a receptor defect intrinsic to the cell, a distinction the authors note matters for treatment, since some LDLR maturation defects can be pharmacologically corrected, positioning FH iPSC-ECs as a variant-matched platform for screening which patients’ mutations are of the correctable type [68]. In diabetic vasculopathy, patients have elevated blood sugar levels, and it was unclear whether endothelial dysfunction was attributable to hyperglycemia or to genetically dysfunctional endothelial cells. Gorashi et al. were able to culture iPSC-ECs from patients with diabetic vasculopathy. They noted that the diabetic iPSC-ECs showed low angiogenic potential, high risk of thrombosis, and increased inflammation, hallmarks of patients in vivo. Notably, they found that raising glucose levels induces a diabetic phenotype in healthy iPSC-ECs but does not affect the already dysfunctional diabetic iPSC-ECs [69].

An important note in translating iPSC-ECs results is that in vivo endothelial cells rely on shear stress from laminar blood flow to express proteins and regulate inflammation. Shear stress is difficult to replicate in vitro and has been seen to reduce inflammation and thrombotic activity, indicating that cells in vitro are shifted toward a phenotype of endothelial dysfunction [70]. Additionally, iPSC-EC differentiation protocols don’t reliably produce ECs with a specific vascular-bed identity, which raises a significant concern in modeling diseases such as FPAH, which rely specifically on pulmonary vasculature [71]. iPSC-derived ECs also display an immature inflammatory phenotype, not upregulating specific proteins, such as E-selectin and VCAM-1, to the extent observed in somatic ECs [63]. Beyond endothelial cells, iPSC technology has also enabled the generation of vascular smooth muscle cells, expanding the ability to model the multicellular processes that regulate vascular function and disease.

#### 2.3.2. Smooth Muscle Cells

Smooth muscle cells (SMCs) exist in the middle of vessel walls, contracting to regulate blood pressure and connecting with extracellular matrix proteins to provide tensile strength to blood vessels. SMCs can be differentiated from either mesodermal or ectodermal lineages, and all lineages can be differentiated into SMCs via TGFβ1 and PDGF-BB treatment [72]. iPSC-SMCs recapitulate in vivo characteristics, with similar contractile protein expression and behavior to primary SMCs [72].

Human SMCs for cell culture are costly, and animal SMCs may not fully represent the human genome, making iPSC-SMCs incredibly useful for in vitro modeling [72]. Hutchinson–Gilford progeria syndrome (HGPS) is a rare genetic disorder characterized by abnormally rapid aging of children and premature death, typically due to cardiovascular disease [57]. HGPS patient-derived iPSC-SMCs, when subjected to hypoxic and mechanically stressful conditions, show nuclear distortion and cellular senescence, mirroring in vivo results. Using this model, researchers knocked down Progerin using short hairpin RNA (shRNA), discovering that the accelerated cell death phenotype of HGPS SMCs is due to progerin accumulation [72].

In Marfan Syndrome (MFS), caused by a variant in the FBN1 gene, iPSC-SMCs from affected patients showed reduced contractility and calcium flux. TALEN-mediated correction of the FBN1 mutation generated a true isogenic control, restoring both contractility and calcium flux phenotypes to normal [72]. A caveat of hiPSC-SMCs is that they are immature compared to quiescent SMCs and therefore do not fully capture the phenotype. Several strategies have been developed to improve maturity, including extended TGFβ1 treatment, prolonged serum culture, MEK inhibitor treatment, and endothelial coculture under flow. However, no consensus has been reached regarding the optimal method [72]. Additionally, the inability to replicate in vivo forces on blood vessels makes it difficult to model diseases such as aortic dissections, which involve stretching and shear stress. 3D culture systems that apply cyclic stretch or flow-based shear stress have been developed to address this, though they remain limited in scope [72]. Although SMCs play a critical role in maintaining vessel integrity and regulating vascular tone, pericyte dysfunction has also been implicated in numerous cardiovascular and microvascular disorders.

#### 2.3.3. Pericytes

Pericytes are mural cells that wrap around the endothelium of capillaries and small vessels, where they regulate vascular permeability, stability, and microvascular contractility. Cardiac pericytes (CPs) are like SMCs, residing on the walls of blood vessels and regulating blood flow. However, CPs typically act on smaller vessels, such as the vasa vasorum and capillaries [73,74]. Primary cardiac pericytes are difficult to obtain, making iPSC cardiac pericytes (iPSC-CPs) of immense importance. A recent cardiac-specific strategy derives pericytes via a stepwise protocol that guides iPSCs through developmental intermediates to a pure epicardial cell population, using stage-specific activation and inhibition of Wnt, BMP, and other morphogens [75]. This staged approach produced substantially more consistent results than an earlier Wnt-only protocol, which yielded variable epicardial induction efficiency from 3–87% and misdirected over 30% of cells toward a fibroblast fate rather than epicardium [75]. Pericyte identity was confirmed by expression of PDGFRβ, NG2 (CSPG4), and CD146 at levels comparable to primary human pericytes [74,75]. IPSC-CPs naturally formed tube structures in vitro, improved the tube formation of iPSC-ECs when mixed in vitro, and enhanced neovessel maturation when co-implanted with iPSC-ECs in vivo [75]. Sunitinib, a drug known to cause cardiotoxicity by targeting coronary microvascular pericytes in mice, was applied to iPSC-CMs and iPSC-CPs; iPSC-CPs showed significantly greater sensitivity to sunitinib-induced death than iPSC-CMs, reproducing the pericyte-selective toxicity seen in vivo [75,76]. A limitation of iPSC-CPs is that while cardiac pericytes originate from epicardial cells, it is still under debate whether other progenitor cell types, such as cardiac neural crest cells, also contribute to cardiac pericytes. A protocol based solely on epicardial differentiation may not capture the full phenotype of true in vivo CPs [77].

### 2.4. Patient-Specific Disease Modeling

Collectively, the ability to generate different cell types from iPSCs has expanded the repertoire of patient-derived cardiovascular cell models. These advances have laid the foundation for patient-specific disease modeling, enabling researchers to investigate how an individual’s unique genetic background influences disease pathogenesis, progression, and therapeutic response. Patient iPSC models can capture and model an organism’s full genomic context, making iPSCs incredibly helpful for the modeling of diseases with a strong genetic underlying cause [78]. This isogenic correction approach has been used to show causality across several inherited cardiovascular diseases; two cases illustrate the method. LQT2 iPSC-CMs demonstrate reduced IKr relative to healthy controls [54]. This finding, though, cannot be considered causal, due to possible epistatic genetic background effects. To address this, Bellin et al. corrected the KCNH2 mutation in patient-derived iPSCs. This experiment found that fixing the KCNH2 mutation normalized IKr density and action potential duration, and that mutating KCNH2 in healthy control cells induced the disease phenotype, thereby isolating KCNH2 as both necessary and sufficient for the LQT2 electrophysiological phenotype [79].

In the case of FPAH, unaffected mutation carriers (UMCs) and FPAH patients similarly had the BMPR2 mutation, indicating that the mutation itself does not determine who becomes affected by FPAH. A study by Gu et al. sought to determine the genetic background noise that was causing FPAH and observed that UMC iPSC-ECs preserved normal adhesion, survival, migration, and angiogenesis because of the upregulation of BMPR2 signaling activators (LRP1 and CAV1) and downregulation of BMPR2 signaling inhibitors (FKBP1A and GREM1). Additionally, the unaffected phenotype had elevated BIRC3, which is independent of the BMPR2 signaling pathway. Upon CRISPR correction of the BMPR2 mutation in FPAH cells, adhesion and survival were restored, confirming that the BMPR2 mutation is sufficient to cause FPAH in the absence of protective regulators [67].

### 2.5. Applications in Drug Discovery and Precision Medicine

One of the most notable uses of iPSCs is to model a patient’s specific response to drug treatments, given the context of their genetic profile, enabling precision treatment based on iPSC modeled drug responses [80]. A common issue in drug development is cardiotoxicity, an adverse effect of medication on the heart’s electrical or physical function, which has led to roughly 30% of potential therapeutics being abandoned in late-stage clinical trials. iPSC-CMs can classify novel drugs as cardiotoxic much earlier in the development process, saving considerable time, capital, and effort [81]. Doxorubicin, widely used in breast cancer treatment, is associated with doxorubicin-induced cardiotoxicity (DIC) in approximately 7–8% of treated individuals. Burridge et al. investigated iPSC differentiated cells from patients who experienced doxorubicin-induced cardiotoxicity and found that the affected patients’ iPSC-CMs experienced greater loss of viability, increased oxidative stress, and impaired calcium handling compared to iPSC-CMs from unaffected individuals. These results have only been confirmed in patients post-DIC and have not been shown to detect DIC [82]. In a patient with LQTS experiencing frequent life-threatening ventricular arrhythmias and defibrillator shocks, alternative treatments were sought to decrease defibrillator shocks and iPSC-CMs were generated from the patient to screen literature-identified candidate drugs, and both verapamil and lidocaine were administered to the cells in vitro. Both drugs were seen to decrease the iPSC-CM’s QT interval, bringing it to levels comparable to those of healthy controls. Verapamil was administered to the patient, who experienced a decreased QT interval and reduced defibrillator discharge frequency, showing the potential to use iPSCs to screen for and predict effective treatments. However, this was in a single patient and cannot yet be generalized to the general population [83].

**Table 1:** Major cardiovascular diseases modeled using iPSC-derived cells.

Disease	iPSC Cell Type	Gene/Model Trigger	Key In Vitro Findings	Ref(s)
Dilated cardiomyopathy (DCM)	iPSC-CM	TTN (truncating)	Impaired myofibril assembly and sarcomere instability; antisense exon-skipping (exon 326) rescued assembly, replicated in animal models	[51]
Hypertrophic cardiomyopathy (HCM)	iPSC-CM	MYH7, MYBPC3	Hypercontractility, increased tension, and delayed relaxation kinetics after CRISPR-induced mutation	[52,53]
Long QT syndrome type 2 (LQT2)	iPSC-CM	KCNH2	60–72% reduction in IKr with delayed repolarization; isogenic KCNH2 correction normalized IKr and action potential duration	[54,79]
Catecholaminergic polymorphic ventricular tachycardia (CPVT)	iPSC-CM	RyR2	Abnormal calcium handling on adrenaline exposure, absent in controls	[55]
Ischemic heart disease (IHD)	iPSC-CM	Hypoxia/reperfusion	Sarcomere damage and cell death under hypoxia; reversible electrophysiological dysfunction (reduced beating frequency, prolonged depolarization, slowed conduction) on reoxygenation	[56]
Heart failure (HF)	iPSC-CM	Endothelin-1 exposure	Increased stress markers (NPPA, NPPB, ACTA1), reduced contractile force, fibrotic and proteostatic dysfunction; stronger induction in 3D organoids	[58]
Familial pulmonary arterial hypertension (FPAH)	iPSC-EC	BMPR2	Impaired adhesion, reduced survival, impaired migration, and disordered angiogenesis; CRISPR correction of BMPR2 restored adhesion and survival	[67]
Familial hypercholesterolemia (FH)	iPSC-EC	LDLR	Reduced LDL uptake and lower mature LDLR intrinsic to the cell, independent of cholesterol exposure	[68]
Diabetic vasculopathy	iPSC-EC	Patient-derived (hyperglycemia)	Low angiogenic potential, elevated thrombotic risk, increased inflammation; dysfunctional cells unresponsive to further glucose elevation	[69]
Hutchinson–Gilford progeria syndrome (HGPS)	iPSC-SMC	LMNA/progerin	Nuclear distortion and senescence under hypoxic and mechanical stress; death driven by progerin accumulation	[72]
Marfan syndrome (MFS)	iPSC-SMC	FBN1	Reduced contractility and calcium flux; TALEN isogenic correction of FBN1 restored both	[72]
Coronary microvascular dysfunction	iPSC-CP	Sunitinib	Pericyte-selective sensitivity to sunitinib-induced death exceeding that of cardiomyocytes	[75,76]

Although iPSC-based models have transformed personalized medicine and drug discovery, traditional 2D cell culture systems are still limited in their ability to recapitulate the complex cellular interactions and tissue architecture of the human heart. To address these limitations, 3D cardiac organoids and spheroids have appeared as advanced platforms that more accurately model cardiac development, physiology, and disease.

### 2.6. Current Limitations of iPSC-Based Cardiovascular Models

Despite their transformative potential, hiPSC-based cardiovascular models retain important biological and technical limitations. Most notably, hiPSC-derived cardiomyocytes (hiPSC-CMs) exhibit a relatively immature phenotype, with fetal-like morphology, sarcomeric organization, electrophysiology, calcium handling, and metabolism. Although prolonged culture, metabolic conditioning, three-dimensional organization, and electromechanical stimulation improve maturation, these approaches do not fully recapitulate adult myocardium [84,85,86,87].

Reproducibility represents a second major challenge. Differentiation efficiency, cellular subtype composition, and maturation vary across hiPSC lines, donors, batches, and protocols, complicating cross-study comparisons. Patient-derived models are additionally influenced by genetic background, while reprogramming can alter age- and environment-associated epigenetic signatures. Genome-edited isogenic controls can help set up variant-specific effects but remain susceptible to clonal and editing-associated variability. Conventional hiPSC cultures also lack the multicellular organization, extracellular matrix, mechanical loading, vascular flow, immune interactions, and neurohumoral signals that shape cardiovascular disease in vivo. Consequently, their ability to model complex and age-dependent disorders or prospectively predict patient-specific therapeutic efficacy and toxicity is still limited. Standardized differentiation and quality-control frameworks, together with validation in diverse patient populations, will therefore be essential for clinical translation. These limitations have accelerated the development of multicellular 3D organoids and engineered cardiac tissues that incorporate increasingly physiological structural and biophysical cues.

## 3. 3D and Engineered Human Cardiovascular Models

Three-dimensional cardiac models include cardiac spheroids, self-organizing organoids, and engineered heart tissues. Although these models are sometimes grouped, they are made differently. Cardiac spheroids are usually formed by combining cardiomyocytes with selected non-cardiomyocyte populations. In comparison, cardiac organoids are created from pluripotent stem cells that differentiate and organize themselves into more complex cardiac structures [88]. Cardiac spheroids and organoids provide several advantages over traditional two-dimensional cultures. Cells in these models can communicate and interact with the surrounding extracellular matrix in all directions. This creates more realistic patterns of metabolism, electrical activity, contraction, and tissue remodeling [89,90]. These models can also be made using patient-specific induced pluripotent stem cells, which makes them useful for studying genetic diseases and differences in individual drug responses [91]. For consistency throughout this review, we use cardiac spheroid to describe three-dimensional aggregates assembled primarily from pre-differentiated cardiovascular cell populations; cardiac organoid to describe pluripotent-stem-cell-derived, self-organizing multicellular structures that reproduce aspects of cardiac development or tissue organization; and engineered cardiac tissue to describe constructs in which cellular organization, geometry, extracellular matrix composition, or mechanical boundary conditions are externally imposed through bioengineering approaches. These categories can overlap experimentally, but the terminology is used according to the dominant mechanism of construct formation.

### 3.1. Development of Cardiac Organoids

Early three-dimensional cardiac models were primarily created by grouping pre-differentiated cardiomyocytes. Researchers later added fibroblasts and endothelial cells to make these spheroids more like human cardiac tissue [89,92]. Common production methods included hanging drops, low-attachment plates, microwells, and rotating culture systems. These spheroids improved cell communication and contractile activity compared with two-dimensional cultures. However, they did not fully reproduce the developmental steps that occur when the human heart forms [88].

Modern cardiac organoids are typically derived from human embryonic or induced pluripotent stem cells. The cells are first grouped into embryoid bodies and then exposed to signals that guide their development. Important pathways include Wnt/β-catenin, Activin/Nodal, bone morphogenetic protein, and fibroblast growth factor signaling [90,93]. By controlling the timing and strength of these signals, researchers can guide the cells through mesoderm formation, cardiac progenitor development, and differentiation into different cardiac cell types. The timing of these signals is especially important. Even small differences in the cell line, aggregate size, culture medium, or length of treatment can change the final organoid [88,92]. This is one reason different laboratories may produce cardiac organoids with different cell types, shapes, and levels of function.

Several important studies have demonstrated how cardiac organoids can organize themselves. Hofbauer and colleagues created scaffold-free “cardioids” that developed chamber-like cavities. These cardioids allowed researchers to study how myocardial and endocardial cells interact during early human heart development [94]. Drakhlis et al. created heart-forming organoids having both cardiac and foregut tissues. This model showed how the developing heart may be influenced by nearby embryonic tissues [95]. Additionally, Lewis-Israeli and colleagues used a three-step Wnt signaling method to generate self-assembling human heart organoids. These organoids developed internal chambers, multiple cardiac cell types, vascular-like networks, atrioventricular features, and electrical activity. Their gene expression patterns also resembled those to fetal human cardiac tissue [96]. Cardiac organoids can be produced through scaffold-free, matrix-supported, or bioengineered methods. Scaffold-free methods allow the cells to organize more naturally and are easier to adapt to multiwell plates. Matrix-supported systems give researchers more control over stiffness and extracellular matrix composition [90,93]. Automated liquid handling, microfabricated wells, chemically defined media, and bioreactors may improve the consistency and scale of organoid production [91,92].

### 3.2. Cellular Composition and Self-Organization

One major benefit of cardiac organoids is that they can hold several cardiac cell types. Depending on the protocol, these may include atrial- and ventricular-like cardiomyocytes, endothelial cells, cardiac fibroblasts, smooth muscle cells, epicardial cells, and endocardial cells [90]. Some newer models also include immune cells, neural cells, or separately produced vascular cells [97]. Each cell population has a different function. Cardiomyocytes produce electrical activity and contraction. Fibroblasts regulate extracellular matrix production, tissue stiffness, and intercellular signaling. Endothelial and endocardial cells help form vascular and chamber-like structures. Epicardial cells also provide signals that support cardiac development and repair [94,96]. Because these cells are cultured together, researchers can study how one cell type affects another. For example, an abnormal cardiac phenotype may result from communication among cardiomyocytes, fibroblasts, and endothelial cells rather than from cardiomyocytes alone. This is one reason multicellular organoids may provide more useful information than cultures containing only cardiomyocytes. Self-organization happens when cells use developmental signals, adhesion molecules, mechanical forces, and extracellular matrix remodeling to arrange themselves into patterns. Cardioids have shown that myocardial and endocardial cells can work together to form chamber-like structures without a predesigned scaffold [94]. Other human heart organoids have developed internal cavities, atrioventricular features, and vascular-like networks when exposed to developmental signals at the correct times [96].

Several methods are used to examine organoid structure and function. These include immunohistochemistry, single-cell sequencing, calcium imaging, optical mapping, microelectrode arrays, metabolic testing, and video-based measurements of contraction. However, cardiac organoids can be difficult to image because they are thick, contain extracellular matrix, and move as they beat [98]. Researchers are therefore using tissue clearing, light-sheet microscopy, two-photon imaging, calcium and voltage indicators, and computer-based image analysis to study their internal structures [98]. Although endothelial cells can form vascular-like networks in cardiac organoids, these networks typically do not carry fluid as real coronary blood vessels do. Oxygen and nutrients must instead diffuse through the organoid. This limits organoid size and can cause hypoxia or cell death in the center of larger organoids [88,90]. However, researchers can also use these oxygen gradients to model ischemic heart disease.

### 3.3. Disease-Modeling Applications

#### 3.3.1. Myocardial Infarction

Myocardial infarction does not affect every part of the heart in the same way. The infarct core, border zone, and remote myocardium experience various levels of oxygen, inflammation, fibrosis, and mechanical stress. Three-dimensional cardiac organoids can reproduce some of these regional differences. Richards and colleagues created cardiac organoids with an oxygen-diffusion gradient and subjected them to noradrenergic stimulation. The resulting organoids contained infarct-, border-, and remote-like regions. They also showed metabolic changes, fibrosis, increased tissue stiffness, abnormal calcium handling, and reduced contraction [88]. The researchers also found that hypoxia increased doxorubicin-related cardiac toxicity, showing that drug toxicity may be different in injured and healthy cardiac tissue [99]. Other multicellular heart organoids have been created using hiPSC-derived cardiomyocytes, fibroblasts, and endothelial cells. When these organoids were exposed to hypoxia and reoxygenation, they reproduced aspects of acute myocardial injury and fibrotic remodeling [100].

#### 3.3.2. Cardiac Fibrosis

Cardiac fibrosis involves fibroblast activation and excessive extracellular matrix production. Over time, this increases tissue stiffness and interferes with the heart’s electrical and mechanical function. Fibrosis can be produced in cardiac organoids using ischemia, transforming growth factor-β, activated fibroblasts, or other profibrotic signals [99,100]. In infarct-like organoids, researchers have seen changes in fibrosis-related genes, increases in activated stromal cells, greater tissue stiffness, abnormal calcium handling, and weaker contraction [99]. Multicellular models can also help researchers understand how cardiomyocyte damage, endothelial dysfunction, fibroblast activation, and extracellular matrix production affect one another during fibrosis [100].

#### 3.3.3. Hypertrophic Cardiomyopathy

Patient-derived hiPSCs can be used to study genetic variants associated with hypertrophic cardiomyopathy. When these cells are placed into three-dimensional tissues, researchers can examine changes in cell size, sarcomere structure, calcium handling, electrical activity, metabolism, and contraction [88]. Buono and colleagues created cardiac organoids from genetically healthy hiPSC lines and lines associated with hypertrophic cardiomyopathy. The resulting organoids showed differences between healthy and cardiomyopathic tissues, supporting their use as three-dimensional models of genetic heart disease [101]. Researchers can also use CRISPR to correct a patient’s mutation or introduce the same mutation into a healthy cell line. These isogenic controls make it easier to determine whether a phenotype is caused by the mutation or by the rest of the patient’s genetic background [88].

#### 3.3.4. Congenital Heart Disease

Cardiac organoids are especially useful for studying congenital heart disease because they model early developmental stages that cannot be observed directly in humans. Heart-forming organoids containing cardiac and foregut tissues have been used to study how interactions between different embryonic tissues affect early heart development [95]. Cardioids have also been used to study cardiac lineage development, chamber formation, and interactions between myocardial and endocardial cells [94]. In another study, NKX2-5-deficient organoids developed abnormal chamber-related features. This provided evidence that genome-edited organoids can connect genetic abnormality to changes in cardiac development [102]. Organoids can also be used to study environmental causes of congenital heart disease. Human heart organoids exposed to conditions associated with pregestational diabetes developed structural and metabolic abnormalities related to congenital heart defects [96]. Low-dose cadmium exposure has also been shown to interfere with mesoderm formation, cardiac differentiation, organoid development, and contraction [103].

### 3.4. Advantages and Limitations

Cardiac organoids provide a more realistic environment than two-dimensional cultures. Their three-dimensional structure supports interactions among different cell types, extracellular matrix remodeling, electrical activity, contraction, and regional injury responses [89,90]. Patient-specific hiPSCs also allow researchers to study inherited diseases and possible differences in drug response [91]. However, cardiac organoids are still simplified models of the human heart. Most organoid cardiomyocytes are more like fetal cardiomyocytes than adult cardiomyocytes. This can make it difficult to model diseases that develop later in life [88,90]. Most models also lack mature blood vessels, normal mechanical loading, autonomic innervation, and complete immune-system involvement. Reproducibility is another concern. Differences in hiPSC lines, aggregate size, passage number, culture media, extracellular matrix, cell ratios, and differentiation timing can produce different results among laboratories [90,92]. The inconsistent use of terms such as spheroid, microtissue, organoid, and engineered heart tissue makes comparisons even more difficult [88].

Imaging also becomes more difficult as organoids thicken and become more complex. Standard microscopy may not show deep structures, internal blood vessels, or small areas of fibrosis. Live imaging must also account for constant contraction, phototoxicity, and limited tissue penetration [98]. Future research should focus on improving cellular maturity, vascular perfusion, immune and neural integration, and standardized production methods. Connecting cardiac organoids to liver, kidney, vascular, or immune tissues may also help researchers model whole-body disease processes [97]. While cardiac organoids have advanced the field by enabling multicellular 3D disease modeling, limitations in tissue organization and maturation have driven the development of engineered heart tissues that more closely mimic the architecture and function of the native heart.

## 4. Cardiac Tissue Engineering

### 4.1. Principles of Cardiac Tissue Engineering

Because of the limited regenerative capacity of adult cardiomyocytes and the shortcomings of conventional in vitro and animal models, there is a growing need for physiologically relevant platforms to study cardiovascular disease and develop novel therapeutic strategies [104,105]. Cardiac tissue engineering has emerged as a promising interdisciplinary field that seeks to recreate the structural and functional properties of native myocardium by combining living cells, biomaterials, and controlled biochemical and biophysical cues [106]. By providing a supportive microenvironment that promotes cell proliferation, attachment, survival, maturation and organization, engineered cardiac tissues offer improved platforms for disease modeling, drug screening, and regenerative medicine. Successful cardiac tissue engineering relies on the integration of appropriate cell sources, biomaterials, structural organization, and dynamic mechanical and electrical stimulation to recapitulate native and cardiac functions.

Replicating the myocardium remains particularly challenging because of its highly organized architecture and coordinated mechanical and electrical activity [107,108]. Consequently, engineered cardiac tissue must be designed to reproduce multiple aspects of the native cardiac microenvironment. Cell selection should reflect the target tissue being modeled, incorporating cardiomyocytes for contractile function alongside supporting cell populations such as fibroblasts, endothelial cells, and vascular smooth muscle cells when appropriate [109]. Biomaterials should be biocompatible and, in many applications, biodegradable while providing structural support that resembles the native extracellular matrix. In addition, engineered constructs should promote vascularization, facilitate electrical coupling between cells, and possess mechanical properties that withstand repetitive contraction while supporting tissue integrity. Together, these design considerations highlight the complexity of engineering functional cardiac tissue.

A variety of engineering strategies have been developed to address these challenges. Scaffold-based approaches use natural or synthetic biomaterials to provide structural support, guide tissue organization, and promote cell adhesion by mimicking features of the native extracellular matrix [110]. Alternatively, scaffold-free approaches rely on cells’ intrinsic ability to assemble into three-dimensional tissues through cell–cell interactions, as described previously [111]. More recently, advances in three-dimensional bioprinting have enabled the precise spatial deposition of cells and biomaterials to fabricate increasingly complex cardiac constructs with improved architectural control [112,113,114]. These engineered tissue platforms complement other advanced cardiovascular models, including cardiac organoids and organ-on-chip platforms discussed elsewhere in this review, while emphasizing the importance of biomaterial selection and construct design in achieving functional tissue maturation and integration. Having proven the design principles underlying engineered cardiac tissues, the choice of scaffold material becomes a critical determinant of construct performance.

### 4.2. Biomaterial-Based Scaffolds

Biomaterial scaffolds serve as the structural foundation of engineered cardiac tissues by recreating key features of native extracellular matrix [109,115]. Beyond providing physical support, scaffolds promote cell attachment, survival, proliferation, and organization while facilitating tissue maturation and integration. An ideal cardiac scaffold must also possess appropriate biodegradability, biocompatibility, and mechanical integrity to support the repetitive contractile forces experienced by the myocardium [116]. In many applications, scaffolds are also designed to facilitate electrical signal propagation to promote synchronized cardiomyocyte contraction. To satisfy these diverse functional requirements, biomaterials used for cardiac tissue engineering generally fall into three broad categories: natural biomaterials, synthetic biomaterials, and decellularized extracellular matrices.

#### 4.2.1. Natural Biomaterials

Natural biomaterials are widely used in cardiac tissue engineering because they closely resemble the native extracellular matrix, providing intrinsic biochemical cues that support cell adhesion, migration, proliferation, and differentiation [117]. Owing to their biocompatibility, bioactivity, and biodegradability, these materials have become attractive to scaffold candidates for regenerative applications. Most natural biomaterials are derived from renewable biological sources such as plants, animals, and microorganisms [116,118]. Natural biomaterials can be broadly classified into two groups: protein-based biomaterials and polysaccharide-based biomaterials [117].

Protein-based biomaterials are composed of amino acid polymers that form fibrous structural networks resembling those of native extracellular matrix proteins [119,120]. Their inherent mechanical strength and bioactivity make them well-suited for supporting tissue formation, wound healing, and cell–matrix interactions. Common examples include collagen, silk, fibrin, gelatin, and fibronectin, which have been incorporated into cardiac scaffolds, hydrogels, and drug delivery systems [119,121,122]. Polysaccharide-based biomaterials are long chains of monosaccharides that are often more hydrophilic and less mechanically robust than proteins [123]. However, they can form gels and films with tunable porosity and are involved in cell signaling and hydration [124]. Alginate, chitosan, hyaluronic acid, cellulose, and dextran are examples of polysaccharide-based biomaterials and have been used in hydrogels for drug delivery and 3D bioprinting bioinks [123,125]. Hybrid scaffolds combine the structural integrity and cell-adhesive properties of protein-based materials with the tunable mechanical properties and hydration capacity of polysaccharides, thereby enhancing controlled drug release, cell proliferation, and scaffold stability [124]. As a result, hybrid biomaterials are increasingly being explored for ischemic tissue repair, vascular regeneration, and engineering cardiac patches. Although natural biomaterials are sustainable and have low environmental impact, they possess relatively weak mechanical strength, batch-to-batch variability, and rapid degradation [117,118]. As a result, synthetic biomaterials are used to address these disadvantages.

#### 4.2.2. Synthetic Biomaterials

Unlike natural biomaterials, synthetic biomaterials are engineered to provide precise control over their mechanical, chemical, and degradation properties [121,126]. These materials possess strong mechanical strength and consistent manufacturing outcomes [127]. This tunability enables researchers to tailor scaffold stiffness, architecture, porosity, and surface chemistry to mimic the native cardiac extracellular matrix better [128]. Because many synthetic polymers lack intrinsic biological signaling domains, they generally exhibit reduced immunogenicity while providing consistent manufacturing and reproducible material properties [127].

Synthetic biomaterials have been incorporated into numerous cardiac tissue-engineering applications, including cardiac patches, injectable hydrogels, vascular grafts, drug-delivery platforms, and bioprinted constructs. For example, they can be used in synthetic hydrogels (polyethylene glycol (PEG)-based copolymers), composite scaffolds (polyurethane (PU), polycaprolactone (PCL), polyvinyl alcohol (PVA), and silicone-based materials), vascular grafts (poly-L-lactic acid (PLLA) or polyglycolic acid (PGA)), and drug delivery systems (poly(lactic-co-glycolic acid) (PLGA) [121,122,129]. However, due to their production methods, synthetic biomaterials have been shown to generate acidic degradation products that alter the pH of surrounding tissue, thereby affecting cell survival and behavior [128]. Additionally, while mechanical and electrical properties can be optimized, they lack natural biochemical cues, which can limit cell differentiation and integration [130].

#### 4.2.3. Decellularized Matrices

Decellularized tissues remove cellular and antigenic content from tissues while preserving native extracellular matrix physicochemical features, and have emerged as a promising scaffold for tissue engineering [130]. Decellularized matrices can be generated from both human and animal tissues while preserving the native extracellular matrix architecture. Decellularization combines physical, chemical, and enzymatic methods to remove cellular material while minimizing disruption of extracellular matrix structure [131]. The main components left in a decellularized matrix include collagen, elastin, fibronectin, laminin, and matricellular proteins, all efficient in providing tensile strength, elasticity, regulating cell behavior, promoting cell adhesion, and binding sites for extracellular matrix structural proteins [117,132,133,134]. Following decellularization, scaffolds can be recellularized with selected cell populations to generate patient-specific or disease-specific tissue constructs for regenerative applications [135]. A popular example of decellularized matrix scaffolds is heart valve replacements with bovine and porcine arteries [136,137]. Although promising and used in standard procedures, decellularized matrices have several unresolved limitations. Decellularization protocols show inconsistent results across groups, resulting in unpredictability in cell removal efficiency while variably preserving extracellular matrix architecture [138]. Additionally, there is variability and uncertainty in biological variability among donors [139]. Continued efforts to optimize and standardize decellularization protocols aim to improve scaffold reproducibility while minimizing extracellular matrix damage and preserving tissue-specific bioactivity. While biomaterial selection provides the structural framework, successful cardiac tissue engineering also depends on recreating the mechanical and electrical environment of the native myocardium (Table 2).

### 4.3. Mechanical and Electrical Conditioning

Although scaffold composition is essential, engineered tissues must also experience the physical cues present within the native myocardium to achieve functional maturation. The heart functions through the coordinated integration of mechanical contraction and electrical conduction, both of which are essential for maintaining efficient cardiac output.

Native myocardial tissue exhibits unique mechanical properties, including elasticity, stiffness, anisotropy, and viscoelasticity, while simultaneously supporting rapid electrical conduction to synchronize contraction [177,178]. Consequently, engineered cardiac tissues must replicate these biomechanical and electrophysiological cues to promote cardiomyocyte maturation, survival, alignment, and synchronized function. Collectively, these approaches enable investigators to evaluate whether engineered constructs reproduce the mechanical behavior necessary for functional cardiac tissue.

#### 4.3.1. Mechanical Stimulation

Mechanical cues play a fundamental role in regulating cardiomyocyte development, maturation, and function. Native myocardium possesses carefully regulated mechanical properties that allow the heart to repeatedly contract, relax, and accommodate cyclical changes in pressure and volume. Among these properties, tissue stiffness, commonly quantified by Young’s modulus, is a critical determinant of myocardial function. Young’s modulus is an important determinant of the heart’s pumping function and the ability of the myocardium to stretch and recoil [177]. In the ventricular myocardium of humans, the average range of Young’s modulus is approximately 10–20 kPa during early diastole and increases to nearly 500 kPa at the end of systole [179,180]. When integrating tissue engineering principles, it is important that the material of interest can match these physical demands. Deviations from native myocardial stiffness can compromise the performance of engineered tissue. Excessively stiff scaffolds may reduce perfusion, impair cell attachment, and generate strain mismatches with host tissue, whereas overly compliant materials fail to provide adequate structural support or efficient force transmission [180,181]. Beyond bulk mechanical properties, dynamic mechanical stimuli, including changes in preload, afterload, or extracellular matrix stiffness, also regulate cardiomyocyte behavior, alignment, proliferation, survival, and mechanotransduction pathways through mechanisms such as the Frank–Starling Law response [182]. This makes physical testing of these materials crucial.

To recreate these native mechanical environments, investigators have developed a variety of natural, synthetic, and hybrid biomaterials that combine bioactive ligands with tunable mechanical properties [183]. Researchers can test their materials using various methods to recreate stress and strain conditions [184,185]. Ex vivo mechanical testing can be performed using uniaxial or biaxial testing to measure the stress–strain relationship, indentation testing to apply a controlled force over a small area to assess compliance, or tensile testing to assess ultimate tensile strength and failure [186,187]. Dynamic testing is used to measure storage and loss of modulus over time or cyclically with repeated strain [186,188,189]. Computational modeling has become a widely used method for stimulating tissue mechanics under physiological loads, using experimental data to calibrate material parameters using finite element analysis [190]. However, mechanical stimulation must also incorporate electrical signaling to synchronize cardiomyocyte activity.

#### 4.3.2. Electrical Stimulation

Native cardiac contraction is initiated through coordinated electrical impulses originating in the sinoatrial node and propagated through the atrioventricular conduction system to synchronize atrial and ventricular contraction, thus setting the resting heart rate between 60–100 beats per minute in adults [191,192]. The conductivity of this entire process ranges from 0.005–0.1 S/m, and materials should match or exceed this to avoid signal delays or blocks [193,194]. Electrocardiograms can record this electrical signal as: P wave (atrial depolarization), QRS complex (ventricular depolarization and contraction), and finally the T wave (ventricular repolarization) [195].

Replicating native electrical signaling remains a major objective in cardiac tissue engineering. It is important that these engineered biomaterials possess both ionic conductivity, which facilitates physiological ion transport and cell signaling, and electronic conductivity, which enhances electrical communication across engineered constructs [194]. A persistent challenge is that scaffold stiffness and electrical conductivity are often inversely related, requiring careful optimization to achieve both mechanical integrity and efficient electrical coupling [196]. Because of this, biomaterials should provide both elasticity and softness to mimic the native myocardium. Researchers utilize conductive plastics and polymers to achieve biological electrophysiology due to their dual iconic and electronic conductivity, ion channel emulation, and biocompatibility [193,197]. These conductive plastics and polymers mimic the action potential waveform through the precise and slow transport of potassium, sodium, and calcium [192]. Additionally, conductive polymers have been integrated into hydrogel-based scaffolds and other biocompatible polymer scaffolds to enhance cell survival, tissue integration, and signal propagation [198,199,200,201]. Methods for testing the electrical stimulation and physiological systems are detailed later in this review, when discussing organ-on-a-chip systems. Together, mechanical and electrical conditioning promote the maturation and synchronization required for the functional maturation of engineered heart tissues. Collectively, these engineering strategies have enabled the development of increasingly sophisticated cardiac constructs with broad applications in regenerative medicine and disease modeling.

### 4.4. Engineered Heart Tissues

Engineered heart tissues (EHTs) are three-dimensional cardiac constructs designed to reproduce selected structural and functional properties of native myocardium. In contrast to self-organizing organoids, EHTs are generally fabricated using predetermined combinations of cardiovascular cells, extracellular matrix components, defined geometries, and mechanical constraints. This controlled architecture provides investigators with greater ability to manipulate cellular composition, tissue alignment, loading conditions, and functional measurements. Most EHT platforms combine cardiomyocytes with a hydrogel containing extracellular matrix proteins such as collagen or fibrin. Cells embedded within the matrix compact and remodel the surrounding material, forming a contractile tissue between anchoring structures. The resulting tension promotes the alignment of cardiomyocytes and the organization of sarcomeres in the direction of the force. Flexible posts commonly serve as both tissue anchors and force sensors, as post displacement during contraction can be used to estimate contractile force.

Human pluripotent stem cell-derived cardiomyocytes have greatly expanded the applications of EHT technology. Tiburcy and colleagues developed a well-defined engineered human myocardium capable of exhibiting measurable force generation and pharmacological responses, demonstrating the feasibility of producing macroscale human cardiac tissues under controlled conditions [202]. Additional EHT systems have incorporated electrical stimulation and mechanical conditioning to generate increasingly mature cardiac phenotypes [87,203].

EHTs can also incorporate multiple cardiovascular cell populations. Fibroblasts contribute to extracellular matrix production and tissue compaction, while endothelial cells and vascular-supporting cells may improve tissue organization and provide paracrine signals. Adjusting the relative proportions of these cell types allows investigators to examine how multicellular interactions contribute to physiological function or pathological remodeling. Incorporation of disease-associated fibroblasts, for example, can be used to study fibrosis, whereas patient-derived cardiomyocytes can reproduce inherited contractile or electrophysiological abnormalities. A major advantage of EHTs is their ability to provide quantitative functional measurements. Contractile force, contraction and relaxation kinetics, beating frequency, calcium handling, conduction velocity, action-potential properties, and responses to electrical pacing can be measured within intact three-dimensional tissue. These endpoints are particularly valuable for cardiovascular disease modeling because many pathological phenotypes arise at the level of integrated tissue function rather than from alterations in a single molecular pathway.

Patient-specific EHTs have been used to investigate inherited cardiomyopathies and metabolic cardiac disorders. For example, engineered tissues generated from genetically defined hiPSC-CMs have demonstrated disease-associated abnormalities in contractility and calcium handling that may be difficult to detect in conventional monolayer culture [204,205,206]. Combining EHTs with isogenic genome-edited controls further enables direct assessment of variant-specific effects while minimizing confounding from genetic background. Nevertheless, engineered heart tissues remain simplified representations of native myocardium. Most constructs lack full cellular diversity, vascular hierarchy, extracellular matrix organization, chamber geometry, innervation, and mechanical loading present in the intact heart. Increasing tissue thickness also limits oxygen and nutrient diffusion. Consequently, improvements in vascularization, perfusion, maturation, and scalable fabrication remain central goals in EHT development.

### 4.5. Three-Dimensional Bioprinting

Three-dimensional bioprinting extends conventional tissue-engineering approaches by enabling controlled spatial deposition of living cells, biomaterials, and signaling components [207,208,209]. Unlike self-assembling systems, in which tissue structure emerges primarily through biological organization, bioprinting allows researchers to define the initial geometry and cellular distribution of a construct using computer-guided fabrication. This spatial control is particularly attractive for cardiac engineering because native myocardium possesses highly organized anisotropic muscle fibers, vascular networks, extracellular matrix architecture, and distinct anatomical regions.

Most cardiac bioprinting strategies use a bioink containing living cardiovascular cells suspended within a printable biomaterial. Bioinks may contain natural materials such as collagen, fibrin, gelatin, alginate, or decellularized extracellular matrix, as well as synthetic or hybrid polymers. The material must satisfy competing requirements: it must be sufficiently fluid for deposition, mechanically stable after printing, cytocompatible, and capable of supporting cell attachment, survival, remodeling, and maturation [210,211]. Several printing approaches have been explored, including extrusion-based printing, inkjet printing, laser-assisted bioprinting, and embedded printing within supportive matrices. Extrusion-based systems are particularly common because they can deposit relatively high cell densities and a broad range of hydrogel formulations. However, printing parameters must be carefully optimized because excessive shear stress during extrusion can reduce cell viability [210,212].

Bioprinting can also reproduce directional organization. Controlled depositions of aligned fibers or microchannels can guide cardiomyocyte orientation and promote anisotropic contraction. Zhang and colleagues developed bioprinted microfibrous scaffolds containing endothelial cells and subsequently added cardiomyocytes, generating aligned myocardial tissue capable of spontaneous and synchronized contraction [209]. Such spatial control may be useful for reproducing the directional electrical and mechanical properties of native ventricular myocardium. Advances in embedded bioprinting have also enabled the printing of soft extracellular matrix proteins that would otherwise deform before stabilization. The Freeform Reversible Embedding of Suspended Hydrogels (FRESH) strategy, for example, uses a temporary support bath to enable high-resolution deposition of collagen-based structures. This approach has been used to reproduce patient-specific anatomical features and fabricate complex cardiovascular structures containing multiscale channels and valve-like geometries [208]. Subsequent work demonstrated FRESH-printed cardiac tubes populated with human stem cell-derived cardiomyocytes and cardiac fibroblasts that remained patent and developed synchronous contractions [213]. Bioprinting may additionally enable personalization of tissue architecture. Patient imaging data derived from magnetic resonance imaging or computed tomography can be converted into digital printing templates, while patient-derived cells and extracellular matrix materials can potentially provide biologically individualized components. Noor and colleagues demonstrated the feasibility of printing thick vascularized cardiac patches using patient-derived cellular and extracellular matrix components, illustrating the potential for personalized cardiac biofabrication [207].

Despite these advances, the fabrication of fully functional human myocardium remains challenging. Increasing printing resolution frequently reduces throughput, while highly printable biomaterials may not provide optimal biological properties. Cell viability can be affected by shear stress, crosslinking conditions, and prolonged fabrication times. Furthermore, reproducing the hierarchical vasculature, cellular density, anisotropic architecture, electrical integration, and mechanical strength of adult myocardium remains difficult. Bioprinting should therefore currently be viewed primarily as a method for controlling tissue architecture and cellular organization rather than as a means of producing complete replacement hearts.

### 4.6. Vascularization Strategies

Vascularization is one of the most important unresolved challenges in cardiac tissue engineering [214,215,216]. Cardiomyocytes have high metabolic requirements and depend on continuous delivery of oxygen and nutrients. In native myocardium, nearly every cardiomyocyte lies near a capillary. In contrast, many engineered tissues rely predominantly on diffusion from the surrounding culture medium. As tissue thickness increases, diffusion becomes insufficient, producing oxygen and nutrient gradients that may lead to impaired function and necrosis [216,217]. One approach to address this limitation is to incorporate endothelial cells into engineered tissues. Endothelial cells can self-organize into vessel-like networks and provide paracrine signals that influence cardiomyocyte maturation and survival [216,218]. Supporting populations such as pericytes, vascular smooth muscle cells, and fibroblasts may further stabilize these endothelial structures. Prevascularization prior to implantation may also facilitate anastomosis to the host vasculature after transplantation. Three-dimensional biomaterials can be designed to promote vascular organization by controlling porosity, stiffness, biochemical signaling, and extracellular matrix composition. Angiogenic factors, such as VEGF, can be incorporated into scaffolds or delivered via engineered cells to stimulate endothelial cell growth. However, simply forming endothelial networks does not ensure functional perfusion. For large, engineered tissues, these networks must develop patent lumens and connect to a fluid-delivery system capable of supporting sustained flow. Microfabrication and bioprinting provide strategies for creating predefined channels within engineered constructs. Sacrificial materials can be printed and later removed to generate hollow vascular conduits, while endothelial cells can be seeded along channel surfaces to produce vascular-like lumens. Bioprinted microvascular networks can simultaneously provide structural organization and improve mass transport across thicker tissues [207,219,220].

Perfusion-based culture systems stand for another important development. Connecting engineered vessels to pumps or microfluidic devices allows culture medium to flow through the construct rather than simply around it. Flow supplies oxygen and nutrients while simultaneously exposing endothelial cells to physiologically relevant shear stress. Functional microvascularization of human-engineered myocardium has been demonstrated in vitro, supporting the feasibility of maintaining vascular networks within contractile cardiac tissue [221]. More recent work has similarly shown that extended in vitro vascularization can improve the subsequent functional performance of engineered human cardiac tissues following implantation [222]. Restoring native vascular complexity, however, remains considerably more difficult than generating isolated channels. Human myocardium contains a hierarchical network ranging from coronary arteries to arterioles and dense capillary beds. Each vascular compartment has distinct cellular, extracellular matrix, mechanical, and hemodynamic properties. Current engineered models generally reproduce only selected components of this hierarchy. Vascularization, therefore, represents both a technical requirement and a biological opportunity. More advanced vascularized tissues can improve cell survival and tissue thickness while simultaneously enabling investigation of endothelial dysfunction, microvascular disease, thrombosis, vascular inflammation, ischemia, and interactions between cardiac and vascular cells. The integration of engineered myocardium with perfusable vascular systems is consequently an important step toward more physiologically relevant cardiovascular disease models.

### 4.7. Applications in Disease Modeling and Regenerative Medicine

One of the primary goals of cardiac tissue engineering is to restore myocardial structure and function following injury through regenerative therapies that integrate biomaterials, living cells, and bioengineering strategies. Currently, researchers are aiming to produce bioengineered cardiac patches that combine regenerative scaffolding and cell delivery [223,224,225,226]. These patches aim to restore contractility and electrical coupling after a myocardial infarction. Despite differences in design, most cardiac patch technologies can be broadly categorized into scaffold-free cellular constructs, cell-free biomaterial scaffolds, and hybrid cell-scaffold systems incorporating hiPSC-derived cardiomyocytes [227,228,229,230]. Although encouraging results have been proven in preclinical studies, limited cell engraftment, vascular integration, and long-term functional survival remain major barriers to clinical translation. Emerging applications to address these shortcomings include personalized medicine, drug screening, and disease modeling. Engineered cardiac tissues also provide physiologically relevant platforms for disease modeling, drug screening, and precision medicine applications. When integrated with complementary technologies such as organ-on-a-chip systems, patient-derived hiPSCs, and AI-based analytical tools, engineered cardiac tissues enable more predictive assessment of disease mechanisms and therapeutic responses [231,232,233]. Collectively, these advances are shifting cardiovascular research away from traditional two-dimensional culture systems toward increasing patient-specific and physiologically relevant experimental models.

Despite substantial progress, several challenges remain before engineered cardiac tissues achieve widespread clinical implementation. Future research is focused on improving tissue complexity, scalability, and clinical translation. A major priority is the incorporation of functional, perfusable vascular networks that improve oxygen and nutrient delivery while promoting cardiomyocyte maturation and long-term tissue survival. Advances in decellularized scaffolds, three-dimensional bioprinting, and hybrid biomaterial systems are bringing the field closer to whole-organ bioengineering, with the long-term goal of generating temporary or permanent bioartificial hearts that overcome donor shortages and immune rejection. AI and machine learning approaches are also emerging as valuable tools for optimizing scaffold design, predicting tissue performance, and developing personalized regenerative therapies [121]. Together, these advances highlight the rapidly evolving nature of cardiac tissue engineering and underscore its potential to transform cardiovascular disease modeling, regenerative medicine, and ultimately clinical care. Building upon these innovations, organ-on-a-chip technologies further enhance physiological relevance by incorporating controlled microenvironments, perfusion systems, and tissue–tissue interactions that more closely mimic human cardiovascular function.

### 4.8. Advantages and Limitations of Cardiac Tissue Engineering

Cardiac tissue engineering bridges the gap between reductionist two-dimensional cultures and the structural and functional complexity of the human myocardium. Three-dimensional organizations promote physiologically relevant cell–cell and cell–matrix interactions, cardiomyocyte alignment, and coordinated force generation, while mechanical and electrical conditioning can enhance maturation. Incorporation of stromal, endothelial, and other non-cardiomyocyte populations further enables investigation of multicellular mechanisms underlying cardiac physiology and disease [32,87,234].

A key advantage of engineered cardiac tissues is the experimental control they offer. In contrast to self-organizing organoids, engineered systems permit defined manipulation of cellular composition, extracellular matrix (ECM), geometry, and mechanical loading, facilitating mechanistic studies of fibrosis, matrix remodeling, biomechanical stress, and cell-specific drug responses [84,87,202,235]. Importantly, these platforms provide quantitative functional endpoints, including contractile force, contraction–relaxation kinetics, conduction, calcium handling, and pharmacological responses, which can reveal phenotypes not captured by molecular profiling alone [32,84,236]. Substantial limitations nevertheless remain. Although tissue engineering improves several features of cardiomyocyte maturation, engineered myocardium generally retains a fetal-like phenotype relative to the adult human heart, with outcomes strongly influenced by cell source, ECM composition, geometry, culture duration, and conditioning protocols [84,202,237]. Vascularization remains a major bottleneck: diffusion limits tissue dimensions, and current endothelial networks or perfusable channels do not fully reproduce the hierarchical coronary vasculature [209,235]. Moreover, most models lack autonomic innervation, circulating immune and endocrine signals, systemic metabolism, and whole-organ hemodynamics, limiting their ability to capture complex cardiovascular phenotypes and systemic drug responses [20,209,235]. Scalability and reproducibility present additional translational challenges. Variability in hiPSC lines, differentiation protocols, cellular ratios, biomaterials, tissue geometry, and functional assays complicates interlaboratory comparisons and highlights the need for standardized benchmarks for structural, electrophysiological, metabolic, and mechanical maturity [36,84,237]. Rather than converging on a single “optimal” model, cardiovascular research will likely adopt complementary platforms matched to specific questions: 2D hiPSC systems for scalable screening, organoids for developmental and multicellular biology, engineered heart tissues for quantitative mechanics, bioprinted constructs for spatial organization, and microphysiological systems for perfusion and hemodynamic control [209,235]. Integration of patient-specific hiPSCs and genome editing with vascular, immune, and neural components, advanced biomaterials, spatial and single-cell profiling, and computational approaches should further increase physiological relevance. This increasing complexity provides a natural rationale for heart-on-chip and multi-organ microphysiological systems, which introduce controlled perfusion, biomechanical forces, and tissue–tissue interactions into next-generation cardiovascular models [20,209].

## 5. Organ-on-a-Chip and Microphysiological Systems

Organ-on-a-chip (OoC) technologies, broadly encompassed within microphysiological systems (MPS), integrate human cells or tissues with microengineered environments to reproduce selected aspects of organ physiology. Cardiovascular MPS combines cardiac or vascular cells with microfluidic perfusion, extracellular matrices, mechanical or electrical stimulation, and functional sensing, enabling precise control of flow, shear stress, oxygen tension, mechanical strain, electrical pacing, and drug exposure [30,36]. These features provide dynamic, human-relevant platforms for cardiovascular disease modeling, mechanistic investigation, and therapeutic evaluation.

### 5.1. Evolution of Cardiovascular Microfluidic Platforms

Cardiovascular microfluidic systems have evolved from simple perfusion chambers into increasingly sophisticated platforms incorporating 3D tissues, endothelialized channels, mechanical deformation, electrical pacing, and integrated sensors [30,234]. Microfluidic control enables continuous delivery of nutrients and drugs while reproducing physiologically relevant flow, shear stress, pressure, and oxygen gradients. Integration of cardiomyocytes with fibroblasts, endothelial cells, vascular smooth muscle cells, or immune populations further permits interrogation of multicellular interactions under controlled biophysical conditions. Embedded electrical, mechanical, and optical sensors enable longitudinal assessment of contractility, electrophysiology, calcium handling, conduction, vascular permeability, and biomarker release without disrupting the tissue [238]. However, device materials and fluidic architecture can introduce experimental artifacts; for example, polydimethylsiloxane can absorb hydrophobic compounds, whereas evaporation, bubbles, and external tubing may alter flow and drug concentrations. Alternative materials and standardized fabrication approaches are therefore being developed to improve reproducibility and pharmacological relevance [30].

### 5.2. Heart-on-a-Chip Systems

Heart-on-a-chip platforms range from patterned cardiomyocyte monolayers to mechanically constrained 3D cardiac microtissues. Whereas 2D configurations facilitate cellular alignment and electrophysiological measurements, 3D systems better reproduce cell–cell and cell–matrix interactions and enable tissue-level measurements of contractile function [30,234]. Mathur et al. developed a hiPSC-derived cardiac MPS that exhibited quantifiable electrophysiological and contractile responses to cardioactive compounds [239]. Marsano et al. subsequently incorporated cyclic mechanical strain into 3D cardiac microtissues, improving tissue organization and functional responses [240], Integrated strain sensors have enabled continuous, non-destructive measurement of cardiac contraction [238]. Increasing cellular complexity further improves physiological relevance. Multicellular heart-on-a-chip systems incorporating hiPSC-derived cardiomyocytes, fibroblasts, and endothelial cells can reproduce vascular responses to flow while enhancing cardiac contractility compared with less complex cultures [241]. Such systems provide an experimental framework for dissecting interactions among myocardial, stromal, and vascular compartments. Heart-on-a-chip technologies are particularly suited to modeling spatially and mechanically heterogeneous disease states. Ischemia-on-a-chip platforms reproduce hypoxia-associated cardiac dysfunction, whereas oxygen gradients can generate distinct healthy, border-zone, and ischemic tissue phenotypes [242,243]. Fibrosis-on-a-chip systems similarly reproduce focal and interstitial remodeling and their differential effects on tissue structure, electrophysiology, and contractility [244]. Chamber-specific atrial and ventricular platforms further enable investigation of region-dependent electrophysiology, arrhythmogenesis, and pharmacological responses [245].

### 5.3. Vessel-on-a-Chip Systems

Vessel-on-a-chip systems recreate key aspects of the vascular microenvironment by combining endothelialized microchannels with controlled perfusion and, where appropriate, vascular smooth muscle cells, pericytes, fibroblasts, or immune cells. These platforms permit precise modulation of shear stress, pressure, oxygenation, and inflammatory stimulus and are therefore particularly valuable for investigating endothelial dysfunction, vascular permeability, inflammation, atherosclerosis, and thrombosis [246].

Multicellular vascular chips have reproduced early features of atherogenesis, including interactions among endothelial cells, vascular smooth muscle cells, monocytes, and macrophages under controlled flow [247]. Thrombosis-on-a-chip platforms similarly enable direct visualization of platelet activation and thrombus formation under defined hemodynamic conditions and have been used to evaluate antithrombotic therapies [248]. Incorporating immune cells can further reveal interactions between vascular inflammation and coagulation [249]. However, vascular phenotypes are highly dependent on anatomical context. Arteries, veins, capillaries, and organ-specific microvascular beds differ in cellular composition, pressure, shear stress, and barrier properties. Appropriate cell identity, vessel geometry, and hemodynamic conditions must therefore be matched to the vascular disease being modeled rather than assuming that a single vessel-on-a-chip configuration represents the entire circulation.

### 5.4. Multi-Organ and Integrated Platforms

Interactions with the liver, kidney, lungs, vasculature, immune system, and other organs shape cardiovascular function and drug responses. Multi-organ MPS addresses this limitation by fluidically coupling tissue-specific compartments, enabling investigation of systemic interactions and organ-dependent drug metabolism. These platforms are particularly relevant for cardiotoxicity studies, as hepatic metabolism can generate active or toxic metabolites that are absent from isolated cardiac models.

Multi-organ systems have been used to simultaneously assess therapeutic efficacy and off-target toxicity across different tissues [250]. Ronaldson-Bouchard et al. interconnected mature human heart, liver, bone, and skin tissues through vascular flow while preserving tissue-specific environments, demonstrating sustained inter-organ communication within a controlled platform [251]. Other vascularized multi-chip systems integrated experimental measurements with computational modeling to predict human pharmacokinetic behavior [252]. Despite their potential, multi-organ systems introduce substantial engineering complexity. Individual tissues require distinct media, flow conditions, metabolic demands, and physiological scaling. Reproducing clinically relevant relationships among tissue mass, fluid volume, drug exposure, and metabolism, therefore, remains a major challenge for translating multi-organ MPS into predictive preclinical platforms. Although cardiovascular MPS have demonstrated increasingly sophisticated control of hemodynamic and tissue-level variables, the strength of evidence supporting specific applications remains heterogeneous. Many studies establish technical feasibility or reproduce selected physiological responses within individual device architectures rather than prospectively demonstrating superior prediction of human clinical outcomes. Comparisons across studies are complicated by differences in cell source, device materials, chamber dimensions, flow rates, mechanical stimulation, sensor platforms, and analytical endpoints. Consequently, demonstration of a disease-associated phenotype within an MPS should be distinguished from evidence that the platform provides reproducible or clinically predictive performance across laboratories.

### 5.5. Drug Screening and Cardiotoxicity Assessment

Cardiovascular MPS provides a particularly attractive platform for therapeutic screening because drug-induced toxicity can be evaluated across multiple functional domains, including electrophysiology, calcium handling, contractility, metabolism, endothelial integrity, and thrombosis. Integrated sensors enable longitudinal assessment before, during, and after drug exposure, allowing transient pharmacological effects to be distinguished from persistent tissue injury [238,239,240]. Importantly, disease context can modify pharmacological responses. Cardiac models exposed to hypoxic or fibrotic environments may display drug sensitivities distinct from those of healthy tissue, emphasizing the value of testing therapeutics under disease-relevant conditions. Vessel-on-a-chip platforms similarly enable assessment of endothelial injury, platelet activation, vascular inflammation, and thrombosis under controlled hemodynamic conditions [247,248].

Integration with patient-specific hiPSCs and genome-edited isogenic controls further creates opportunities for precision pharmacology by linking genotype to drug response. However, routine translational implementation will require standardized cell sources, device fabrication, fluidic conditions, sensor calibration, and analytical endpoints, together with benchmarking against human clinical data [30,234]. Standardization remains a recognized barrier to broader adoption of heart-on-chip systems. Overall, cardiovascular MPS bridge the gap between reductionist in vitro cultures and systemic physiology by integrating human cells with controlled biochemical, mechanical, electrical, and hemodynamic cues. Their convergence with patient-specific stem cells, genome engineering, and computational analysis offers a promising framework for mechanistic disease modeling and therapeutic development. In parallel, precise manipulation of disease-associated genetic variants has become essential for establishing causal genotype–phenotype relationships, motivating the integration of CRISPR-based genome engineering into next-generation cardiovascular models.

## 6. CRISPR-Based Genome Engineering in Cardiovascular Disease

Genome engineering has transformed cardiovascular research by enabling causal interrogation of disease-associated variants, generation of genetically defined human models, systematic functional screens, and development of targeted therapeutic strategies [34,253,254]. CRISPR-Cas systems are particularly powerful when integrated with patient-derived hiPSCs, engineered tissues, organoids, microphysiological systems, and single-cell technologies, providing a direct link between genetic variation, cellular phenotype, and cardiovascular disease mechanisms.

### 6.1. CRISPR–Cas9 Gene Editing

CRISPR systems were initially identified as repetitive genomic elements in bacteria and were subsequently recognized as components of adaptive microbial immunity [255,256]. Their adaptation of programmable genome editing established CRISPR-Cas9 as a versatile platform for targeted genetic manipulation. In its canonical form, a guide RNA directs Cas9 to a complementary genomic sequence, where Cas9 generates a DNA double-strand break (DSB) [257,258]. Repair through non-homologous end joining (NHEJ) frequently generates insertions or deletions that disrupt gene function, whereas homology-directed repair (HDR) can introduce defined sequence changes using a donor template [259]. Because HDR is cell-cycle-dependent and generally less efficient than NHEJ, precise editing can remain challenging, particularly in differentiated or non-dividing cardiovascular cells. Compared with earlier programmable nucleases, including zinc-finger nucleases and transcription activator-like effector nucleases (TALENs), CRISPR-Cas systems offer greater flexibility and scalability for genetic manipulation [260,261]. In cardiovascular research, CRISPR editing of hiPSC-derived cardiomyocytes and vascular cells enables introduction or correction of disease-associated variants, functional validation of candidate genes, and generation of genetically defined models of cardiomyopathies, arrhythmias, congenital heart disease, and vascular disorders [262,263]. These approaches are particularly valuable for establishing causal genotype–phenotype relationships that cannot be resolved through association studies alone.

Important limitations nevertheless remain. Unintended editing can occur at off-target sites, while DSBs may induce large deletions, chromosomal rearrangements, DNA damage responses or p53-dependent cell-cycle arrest [264,265]. Editing efficiency is also highly dependent on cell type, target locus, and delivery strategy. In vivo cardiovascular applications face additional challenges associated with tissue-specific delivery, immune responses, editing efficiency, and long-term safety. These limitations have accelerated the development of editing strategies that can introduce precise genetic changes without requiring DSBs.

### 6.2. Base Editing and Prime Editing

Base and prime editing expand the precision of CRISPR-based genome engineering while reducing dependence on DSB-mediated DNA repair. Base editors combine a catalytically impaired Cas protein with a nucleotide-modifying enzyme, enabling targeted single-nucleotide conversions without conventional DSB formation [266,267]. Cytosine and adenine base editors collectively enable the four transition substitutions—C→T, G→A, A→G, and T→C—and can function in both dividing and non-dividing cells. This capability is particularly relevant to cardiovascular genetics, where numerous pathogenic variants are single-nucleotide substitutions.

Prime editing provides broader sequence versatility by coupling a Cas9 nickase to an engineered reverse transcriptase and using a prime-editing guide RNA (pegRNA) that specifies both the genomic target and desired edit [268]. This “search-and-replace” strategy can introduce targeted substitutions, small insertions, and deletions without requiring a DSB or exogenous donor DNA. Prime editing, therefore, expands the spectrum of potentially correctable variants beyond those accessible to conventional base editors. Both technologies have emerging applications in cardiovascular disease modeling and therapeutic development. Base editing has shown promise for correcting pathogenic variants associated with inherited cardiomyopathies and for modifying genes involved in cardiovascular risk [269,270]. Prime editing similarly provides a strategy for modeling or correcting disease-associated variants that cannot readily be addressed through transition base editing. Its application to disorders such as Duchenne muscular dystrophy, in which progressive skeletal muscle disease is frequently accompanied by cardiomyopathy, further illustrates its potential relevance to cardiovascular medicine [271]. However, DSB-free does not imply risk-free editing. Base editors can generate bystander or unintended nucleotide changes, whereas prime editing may produce incomplete or undesired editing products and generally exhibits context-dependent efficiency. For both platforms, efficient delivery to cardiovascular tissues, editing specificity, immunogenicity, and long-term genomic safety remain major translational challenges [272,273].

### 6.3. CRISPR-Based Functional Genomics

Whereas conventional genome editing typically interrogates individual genes or variants, CRISPR-based functional genomics enables systematic perturbation of hundreds to thousands of genes in parallel. Loss-of-function (CRISPR–Cas9) screens identify genes required for a phenotype, whereas CRISPR interference (CRISPRi) and CRISPR activation (CRISPRa) use catalytically inactive Cas proteins coupled to transcriptional regulators to repress or activate gene expression without altering the underlying DNA sequence [274,275,276]. Screens can be performed in pooled or arrayed formats. Pooled libraries provide high-throughput discovery by introducing large guide-RNA collections into a cell population and identifying perturbations enriched or depleted following phenotypic selection [274,277]. Arrayed approaches spatially separate individual perturbations, helping direct genotype–phenotype assignment and more complex functional measurements, albeit at lower throughput. These complementary strategies enable systematic identification of genetic regulators of cell survival, differentiation, stress responses, and disease-associated phenotypes.

In cardiovascular models, CRISPR screens have been applied to identify regulators of cardiomyocyte differentiation and maturation, pathological remodeling, fibrosis, cellular stress, and therapeutic response [278,279]. Their integration with advanced human models substantially extends this capability. Organoids and engineered tissues enable candidate genes to be evaluated in multicellular, mechanically relevant environments. In contrast, microphysiological systems permit genetic perturbations to be examined under controlled flow, mechanical stress, or drug exposure. Particularly powerful is the convergence of CRISPR screening with single-cell omics. Perturbation-based single-cell approaches link individual genetic manipulations to transcriptomic states, enabling simultaneous interrogation of gene function across heterogeneous cell populations [275]. Integration with spatial and multimodal profiling could further reveal how genetic perturbations reshape cell states and intercellular communication within diseased cardiovascular tissues. These approaches shift cardiovascular functional genomics from candidate-gene studies toward systematic mapping of causal disease networks.

### 6.4. Generation of Isogenic Disease Models

One of the most important applications of CRISPR in cardiovascular research is the generation of isogenic disease models. Patient-derived hiPSCs retain the donor’s genetic background, but comparisons with unrelated healthy controls are confounded by extensive interindividual genetic variation. Isogenic models minimize this variability by comparing cells that share an otherwise closely matched genetic background but differ at the disease-associated locus [254,280]. Two complementary strategies are commonly employed. Pathogenic variants can be corrected in patient-derived hiPSCs to determine whether restoration of the reference sequence rescues the disease phenotype. Conversely, candidate variants can be introduced into control hiPSCs to determine whether they reproduce the phenotype. Bidirectional editing-phenotypic rescue following correction, together with phenotypic induction following variant introduction, provides particularly strong evidence for variant causality. This strategy has been extensively applied to inherited cardiovascular disorders. Correction of variants affecting genes such as SCN5A, KCNH2, MYBPC3, and TBX20 has been used to rescue disease-associated electrophysiological, contractile, or developmental phenotypes [79,281,282]. Conversely, the introduction of variants in genes, including KCNH2 and ALPK3, has enabled direct characterization of their functional consequences [283,284].

Isogenic models nevertheless require careful interpretation. Clonal selection, unintended genomic alterations, and differences acquired during cell expansion or differentiation can introduce variability independent of the intended edit. Multiple independently edited clones, appropriate unedited controls, genomic quality control, and phenotypic replication are therefore essential. When combined with these safeguards, isogenic hiPSC models provide a powerful framework for distinguishing causal variants from background genetic effects and for linking genotype to therapeutic response.

### 6.5. Therapeutic Applications and Translational Challenges

CRISPR-based technologies are increasingly extending from mechanistic disease modeling toward therapeutic genome editing. Cardiovascular applications broadly include the correction of pathogenic variants, the disruption of disease-promoting genes, the modulation of protective pathways, and the validation of genetically supported therapeutic targets. These approaches are being investigated across inherited cardiomyopathies, arrhythmia syndromes, congenital cardiovascular disorders, and lipid-associated disease [285,286,287]. A particularly attractive therapeutic strategy is the modification of genes that regulate circulating cardiovascular risk factors. Unlike conventional pharmacotherapy, which generally requires repeated administration, durable somatic genome editing could produce sustained therapeutic effects after a single intervention. Base- and CRISPR-based approaches targeting hepatic regulators of lipid metabolism, therefore, illustrate how genome engineering may extend beyond rare monogenic disorders toward more prevalent cardiovascular diseases. However, the risk–benefit threshold for permanent editing is substantially higher when applied to common, clinically manageable conditions. Several barriers remain before cardiovascular genome editing can achieve widespread clinical translation. Efficient and tissue-selective delivery is particularly challenging because the heart contains multiple cell populations and numerous terminally differentiated cardiomyocytes. Viral vectors can provide efficient delivery but are constrained by cargo capacity, immunogenicity, and challenges associated with repeat dosing. In contrast, non-viral approaches may offer greater flexibility but currently exhibit variable efficiency across cardiovascular tissues. Off-target editing, unintended on-target alterations, immune responses, durability, and long-term safety will require rigorous evaluation. Ethical considerations should also distinguish somatic from heritable germline genome editing. Somatic editing targets cells within an individual and is being developed therapeutically for multiple diseases, whereas heritable editing of embryos or germ cells raises fundamentally different ethical and regulatory concerns because genomic alterations could be transmitted to later generations [288,289]. These issues should not be conflated when evaluating the translational potential of cardiovascular genome engineering. From a regulatory perspective, therapeutic cardiovascular genome editing is currently considered within the broader framework governing human gene therapies and genome-editing products. Somatic editing requires rigorous characterization of product identity, delivery, editing efficiency, unintended on- and off-target genomic alterations, immunogenicity, biodistribution, durability, and long-term safety. Because cardiovascular interventions may produce permanent genomic modifications in long-lived cells, extended follow-up and post-treatment surveillance are particularly important. Regulatory assessment is therefore substantially different from the ethical and regulatory considerations surrounding heritable germline editing, which is not the therapeutic focus of the approaches discussed here.

Future progress will increasingly depend on integration with the other next-generation technologies discussed in this review. Single-cell and spatial multi-omics can define the molecular consequences of genetic perturbation. At the same time, advanced human tissue models provide physiologically relevant environments in which candidate variants and therapeutic edits can be validated. AI may further improve guide-RNA design, predict editing outcomes and off-target activity, and prioritize disease-associated variants for functional interrogation [290]. Together, these approaches position genome engineering not as an isolated technology but as a central component of an integrated framework connecting genotype, mechanism, therapeutic target, and patient-specific response (Table 3). Cardiovascular genome-editing studies substantially different levels of evidence, from isogenic disease modeling in hiPSC-derived cells to preclinical therapeutic editing. Correction of a cellular phenotype provides strong evidence for variant causality but does not by itself establish therapeutic feasibility. Translation also requires efficient tissue-selective delivery, clinically meaningful editing efficiency, durability, assessment of unintended on- and off-target alterations, immunogenicity, and long-term safety. These distinctions are particularly important when extrapolating from cellular proof-of-concept studies to potential therapeutic applications.

## 7. Limitations, Reproducibility, and Translational Failure

A balanced assessment of next-generation cardiovascular models also requires consideration of situations in which increasing model complexity has not translated into improved robustness or clinical prediction. hiPSC-derived cells can display substantial line-to-line, clone-to-clone, and batch-to-batch variability, and maturation protocols do not consistently produce adult-like phenotypes. Organoid self-organization can generate heterogeneous cellular compositions and structures, complicating reproducibility across experiments and laboratories. Engineered tissues may improve functional maturation while remaining limited by diffusion, incomplete vascularization, and protocol-dependent mechanical properties. Likewise, organ-on-chip systems can reproduce selected physiological forces but may introduce device-specific artifacts and substantial variability associated with fabrication, materials, fluidics, and sensor calibration. These limitations illustrate an important distinction between biological sophistication and validated predictive performance. A model that reproduces a known phenotype retrospectively is not necessarily able to predict therapeutic efficacy or toxicity prospectively. Clinical readiness should therefore be evaluated using independent replication, interlaboratory benchmarking, blinded testing with reference compounds or phenotypes, and prospective comparison with human outcomes rather than physiological resemblance alone.

## 8. Comparative Evaluation and Selection of Cardiovascular Disease Models

No single cardiovascular model simultaneously maximizes physiological relevance, experimental throughput, reproducibility, scalability, and clinical predictive value. Model selection should therefore be guided by the biological or translational question rather than by technological complexity alone. Conventional two-dimensional cultures remain advantageous for high-throughput mechanistic studies and initial compound screening because they are relatively inexpensive, scalable, and experimentally accessible, but they incompletely reproduce tissue architecture, multicellular interactions, and physiological mechanical cues. Patient-derived hiPSC models provide human genetic context and are particularly valuable for investigating inherited disease mechanisms, genotype–phenotype relationships, and interindividual drug responses; however, cellular immaturity and line-to-line and batch-to-batch variability remain important limitations. Three-dimensional spheroids and organoids provide greater multicellular and architectural complexity and are particularly useful for investigating developmental processes, cell–cell interactions, fibrosis, and disease phenotypes that depend on tissue organization. Their increased physiological relevance is accompanied by lower throughput, greater variability, and challenges in controlling cellular composition and maturation. Engineered cardiac tissues provide greater experimental control over geometry, extracellular matrix composition, mechanical loading, electrical stimulation, and functional measurements such as contractile force, making them particularly suitable for studies of myocardial biomechanics, maturation, and functional drug responses. However, their fabrication is more technically demanding and expensive, and vascularization and adult-like maturation remain incomplete. Cardiovascular microphysiological and organ-on-chip systems are particularly advantageous when the research question depends on dynamic flow, shear stress, mechanical strain, tissue–tissue communication, or pharmacokinetic interactions. These systems can reproduce physiological cues that are difficult to model in static cultures but currently have lower throughput and greater device-to-device and laboratory-to-laboratory variability. Standardization of fabrication, fluidics, sensors, and analytical endpoints remains necessary before their predictive performance can be compared reliably across laboratories. Consequently, model complexity should be increased only when required by the biological question. High-throughput target identification and early toxicity screening may be appropriately performed using standardized two-dimensional hiPSC-derived cells, whereas investigation of multicellular remodeling may require organoids or engineered tissues. Questions involving contractile mechanics are better addressed using engineered heart tissues, while vascular flow and endothelial shear responses favor vessel-on-chip approaches. Patient-specific disease modeling benefits from hiPSC-derived models combined with isogenic genome editing, whereas assessment of systemic pharmacology may require interconnected multi-organ microphysiological systems. In many applications, a tiered strategy in which findings progress from scalable reductionist models to increasingly complex human-relevant systems may provide a more efficient and rigorous pathway toward clinical translation.

## 9. Conclusions and Future Perspectives

Cardiovascular disease modeling is moving from reductionist systems toward increasingly integrated, human-relevant platforms. hiPSC-derived cells provide patient-specific genetic context, organoids and engineered tissues reproduce aspects of multicellular organization and function, microphysiological systems introduce physiological forces and inter-organ interactions, and CRISPR, multi-omics, and computational approaches enable increasingly detailed mechanistic interrogation. These technologies are best viewed as complementary components of an experimental continuum rather than replacements for conventional models. Despite substantial progress, greater biological complexity does not necessarily ensure greater clinical predictivity. Cellular immaturity, incomplete vascularization, limited immune and neural integration, batch-to-batch variability, device-specific fabrication, and non-standardized functional endpoints continue to affect reproducibility and cross-study comparison. Many advanced models also remain supported primarily by proof-of-concept studies or relatively small patient cohorts, with limited prospective validation against clinical outcomes. Standardization and scalability will therefore be essential for translation. Consensus criteria are needed for cell identity and maturity, tissue composition and function, microphysiological operating conditions, genome-editing quality control, and experimental reporting. Interlaboratory benchmarking, reference standards, and validation against human tissues and clinical outcomes will be important for establishing reproducibility and predictive value. At the same time, increasing model complexity generally increases cost, technical burden, and fabrication time while reducing throughput, requiring a balance between physiological fidelity and practical scalability. Regulatory acceptance will depend on reproducible, context-specific predictive performance rather than on physiological resemblance alone. Advanced human-relevant models are therefore likely to complement established preclinical approaches while qualification frameworks continue to develop. Genome-editing applications also require careful evaluation of delivery, unintended genomic alterations, immunogenicity, durability, and long-term safety. Ultimately, the greatest translational value may arise from integrating these technologies. Combining patient-derived cells, genome editing, engineered tissues, microphysiological systems, multi-omics, and computational analysis may provide a more complete link among genotypes, disease mechanisms, therapeutic responses, and clinical outcomes. Standardized and clinically benchmarked integration of these platforms will be critical for advancing predictive cardiovascular modeling and precision medicine.

## Figures and Tables

**Figure 1:**
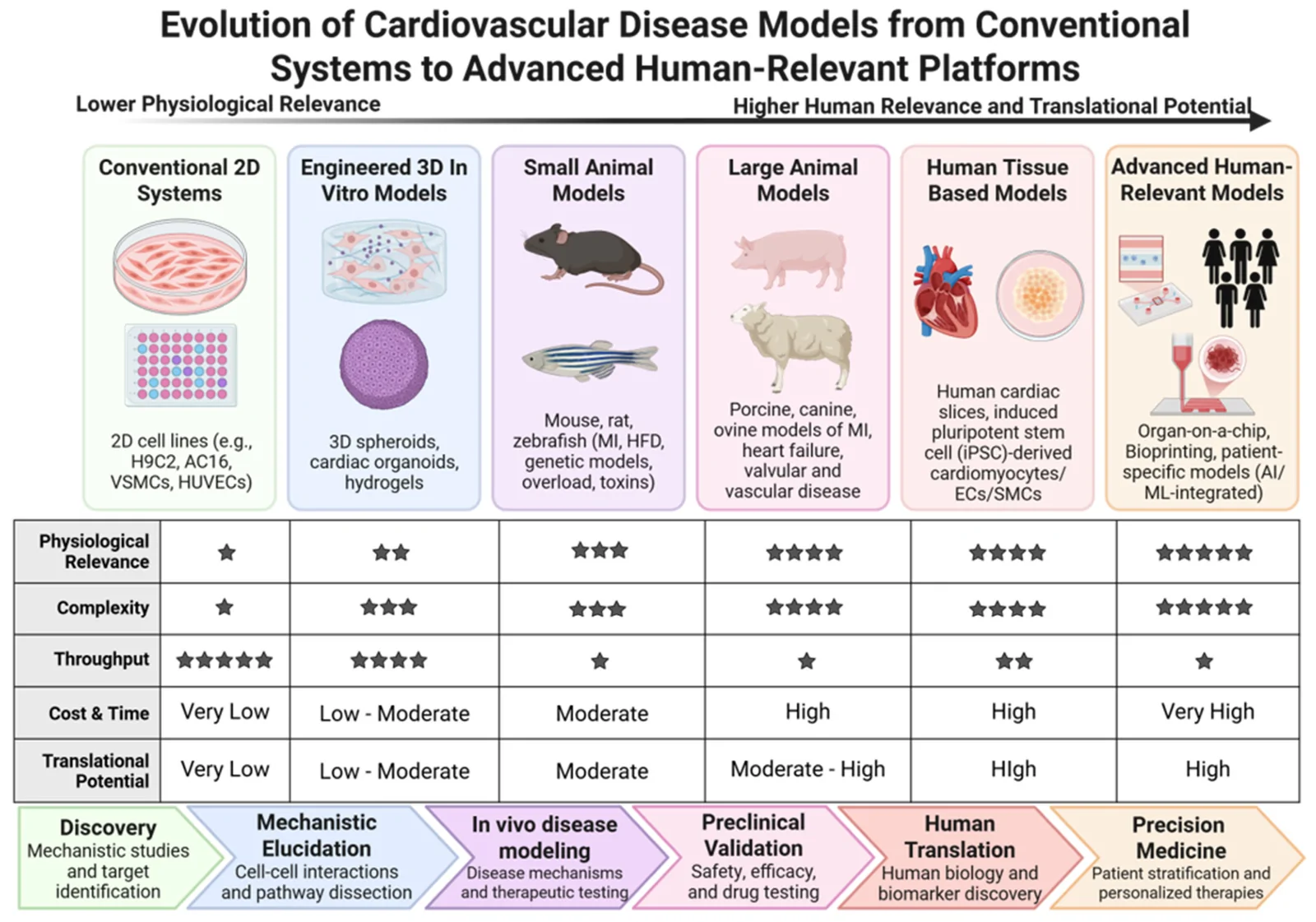
Evolution of cardiovascular disease models toward advanced human-relevant platforms. Cardiovascular modeling spans conventional 2D cultures, engineered 3D systems, small- and large-animal models, human tissue-based models, and advanced human-relevant platforms, with increasing physiological complexity and translational relevance generally accompanied by reduced throughput and greater cost and experimental demands. Collectively, these complementary platforms support the continuum from mechanistic discovery and in vivo validation to human translation and precision medicine. Star ratings represent qualitative, illustrative comparisons among broad model classes rather than validated quantitative scores. Ratings were assigned based on the relative degree of physiological complexity, experimental throughput, resource and time requirements, and current translational applicability described in the literature and discussed throughout this review. These ratings are intended as a conceptual guide for model selection; individual implementations may differ substantially according to cell source, protocol, disease context, and experimental design. (The figure was created with BioRender.com).

**Figure 2:**
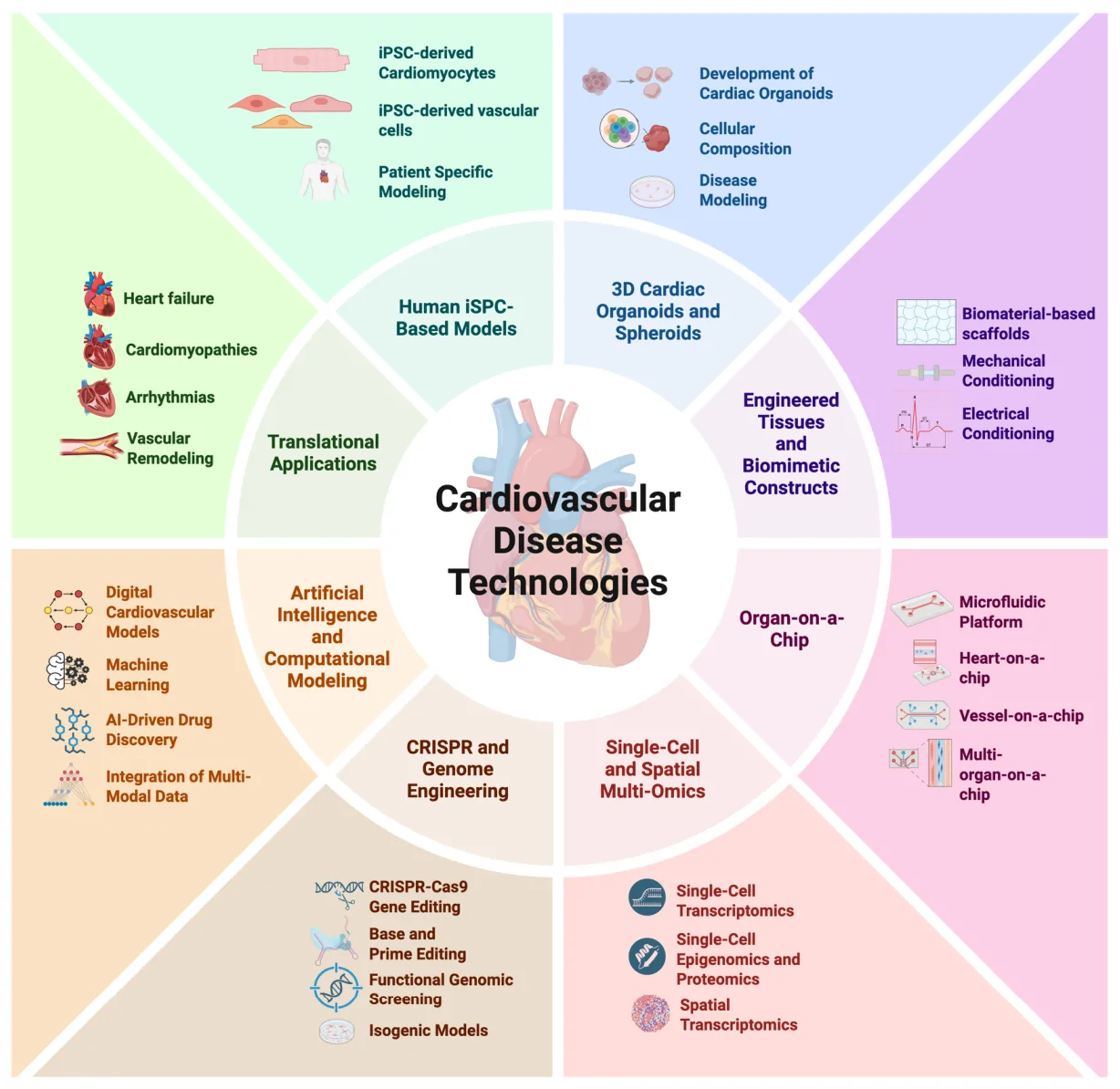
Emerging technologies for human cardiovascular disease modeling. Overview of complementary platforms spanning hiPSC-based models, cardiac organoids and spheroids, engineered tissues, organ-on-chip systems, single-cell and spatial multi-omics, genome engineering, and AI-enabled computational modeling. Together, these technologies integrate human-specific cellular, molecular, biomechanical, and computational information to improve disease modeling, therapeutic discovery, and translational cardiovascular research. (The figure was created with BioRender.com).

**Table 2:** Biomaterials commonly used in engineered heart tissues and their current applications.

		Application	Reference
Natural Biomaterials	Alginate	Cardiac Patches, Encapsulation Injections, Scaffolds	[140,141,142]
	Collagen	Cardiac Patches, Film, Hydrogels	[143,144,145,146]
	Gelatin	Cardiac Patches, Scaffolds	[141,147,148]
	Fibrin	Cardiac Patches, Hydrogels, Scaffolds	[149,150,151]
	Chitosan	Cardiac Patches, Hydrogels, Grafts	[152,153,154]
	Decellularized Matrices	Cardiac Patches, Scaffolds	[155,156,157]
Synthetic Biomaterials	PCL	Cardiac Patches, Scaffolds, Grafts	[147,153,158]
	PVA	Cardiac Patches, Scaffolds, Hydrogels	[159,160,161,162]
	PU	Cardiac Patches, Films, Scaffolds	[161,163,164]
	PEG	Cardiac Patches, Hydrogels	[165,166,167]
	PLGA	Cardiac Patches, Nanoparticles	[168,169,170]
	PGA	Grafts	[171,172,173,174]
	PLLA	Grafts, Occluder	[171,175,176]

**Table 3:** Examples of CRISPR-enabled cardiovascular disease models.

Disease	Target Gene	Model System	CRISPR Application	Citation
Hypertrophic cardiomyopathy	MYBPC3	Human iPSC-derived cardiomyocytes	Isogenic model	[291]
Hypertrophic cardiomyopathy	TNNT2	Human iPSC-derived cardiomyocytes	Isogenic model	[292]
Dilated cardiomyopathy (DCM)	LMNA	Patient-derived iPSC-derived cardiomyocytes	Isogenic model	[190]
Cardiomyopathy	ALPK3	Human iPSC-derived cardiomyocytes, knockout transgenic mouse model	Isogenic introduction model and loss-of-function	[284]
PRKAG2 cardiomyopathy	PRKAG2	Knock-in and transgenic mouse models	Loss-of-function	[293]
Left Ventricular Non-Compaction cardiomyopathy	TBX20	Patient-derived iPSC-derived cardiomyocytes	Isogenic correction model	[281]
Brugada syndrome	SCN5A	Human iPSC-derived cardiomyocytes	Isogenic model	[294]
Long-QT Syndrome	KCNH2	Patient-derived human iPSC-derived cardiomyocytes	Isogenic correction model	[79]
Long-QT Syndrome	KCNH2	Patient-derived human iPSC-derived cardiomyocytes	Isogenic introduction model	[283]
Arrhythmogenic cardiomyopathy	SCN5A	Patient-derived hiPSC-derived cardiomyocytes	Isogenic model	[282]
Arrhythmogenic right ventricular cardiomyopathy	PKP2	Cardiac-specific PKP2-deficient mouse model	Loss-of-function	[295]
Hypercholesterolemia	PCSK9	Mouse liver (in vivo)	Loss-of-function	[296]
Hypercholesterolemia/LDL uptake disorder	TAGLN	HepG2 hepatocyte cell line	Genome-wide perturbation/Pooled screen	[297]
Transthyretin amyloidosis	TTR	Mouse and rat liver (in vivo)	Loss-of-function	[298]

## Data Availability

No new data were created or analyzed in this study. Data sharing is not applicable to this article.
